# Development of Organs-on-Chips and Their Impact on Precision Medicine and Advanced System Simulation

**DOI:** 10.3390/pharmaceutics15082094

**Published:** 2023-08-07

**Authors:** Ying Luo, Xiaoxiao Li, Yawei Zhao, Wen Zhong, Malcolm Xing, Guozhong Lyu

**Affiliations:** 1 Burn & Trauma Treatment Center, The Affiliated Hospital of Jiangnan University, Wuxi 214000, China; 7222808005@stu.jiangnan.edu.cn (Y.L.); lxx19971014@163.com (X.L.); 2 Engineering Research Center of the Ministry of Education for Wound Repair Technology, Jiangnan University, Wuxi 214000, China; 3 Wuxi School of Medicine, Jiangnan University, Wuxi 214000, China; 4 Department of General Surgery, Huai’an 82 Hospital, Huai’an 223003, China; 5 Department of Biosystems Engineering, University of Manitoba, Winnipeg, MB R3T 2N2, Canada; zhaoy13@myumanitoba.ca (Y.Z.); wen.zhong@umanitoba.ca (W.Z.); 6 Department of Mechanical Engineering, University of Manitoba, Winnipeg, MB R3T 2N2, Canada; 7 National Research Center for Emergency Medicine, Beijing 100000, China

**Keywords:** organs-on-chips, drug screening, ADME (absorption, distribution, metabolism, and excretion), maternal–foetal interface, personalised treatment, bone marrow-on-a-chip, AngioChip

## Abstract

Drugs may undergo costly preclinical studies but still fail to demonstrate their efficacy in clinical trials, which makes it challenging to discover new drugs. Both in vitro and in vivo models are essential for disease research and therapeutic development. However, these models cannot simulate the physiological and pathological environment in the human body, resulting in limited drug detection and inaccurate disease modelling, failing to provide valid guidance for clinical application. Organs-on-chips (OCs) are devices that serve as a micro-physiological system or a tissue-on-a-chip; they provide accurate insights into certain functions and the pathophysiology of organs to precisely predict the safety and efficiency of drugs in the body. OCs are faster, more economical, and more precise. Thus, they are projected to become a crucial addition to, and a long-term replacement for, traditional preclinical cell cultures, animal studies, and even human clinical trials. This paper first outlines the nature of OCs and their significance, and then details their manufacturing-related materials and methodology. It also discusses applications of OCs in drug screening and disease modelling and treatment, and presents the future perspective of OCs.

## 1. Introduction

**Figure 1:**
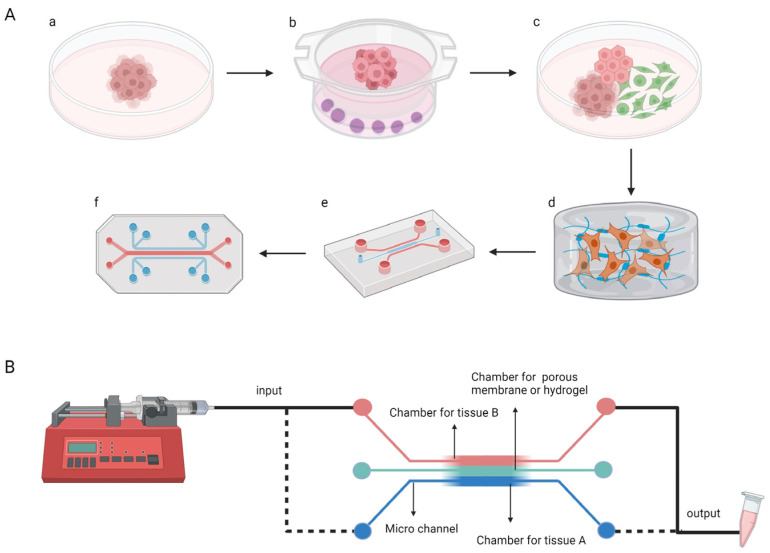
Brief introduction to OCs. (**A**) Drug screening cell models: (**a**) Cells cultured in 2D. (**b**,**c**) Several types of cells cultured separately and then assembled together. (**d**) Cells cultured in a 3D matrix. (**e**,**f**) Cells cultured in a microfluid system and distributed by design. (**B**) Primary composition of OCs, including a microfluidic system and cell culture chambers.

Time and economic challenges limit the use of disease modelling for better treatment. Current animal models allow us to understand pathophysiology and drug screening; however, inconsistencies in findings between animal models and human trials are constantly observed. Many drugs have been screened in vitro and then by using animals; however, when drugs enter the clinical trial stage, they are often suspended due to inadequate efficacy and unexpected side effects [1,2]. Therefore, ideal models and test platforms for better prediction of human responses are urgently required. Current platforms for drug efficacy and toxicity evaluation and disease modelling generally fall into the categories of cell lines, tissue/organ cultures in vitro, organoids, and organs-on-chips (OCs), as shown in Figure 1A. Cell lines are quick and convenient to culture, but cannot mimic numerous phenotypes of cells and tissues in the human body and lack host immunity and tissue- and organ-level responses [3,4]. Although culture methods of in vitro human tissues/organs could produce a highly relevant model for our body system, which is a reasonable approach for various tests, tissue/organs cultured in vitro do not survive well and are expensive; there is also a short supply of their sources. Our body is a combination of complex systems, but human tissues and organs cultured in vitro are independent of those in living organisms. Thus, they cannot be used to study coherent host reaction mechanisms. In addition, large individual differences and poor reproducibility in experimental results limit their in-depth study. Organoids have advanced significantly in recent years. They imitate the growth and interaction of tissues and organs by coculturing various types of cells to form functional tissues with a certain structure, eventually resulting in cellular diversity [5]. Organoids are generalised by the free or well-ordered organisation of cells, and the final formation of the structured tissue is highly correlated to some functions of human organs and tissues [6], but they always neglect the microenvironment, including oxygen gradients and pressure differences, and have insufficient dynamic signals such as those of air flow and blood flow. These signals are crucial for simulating human physiology [7,8,9].

OCs, also known as micro physiological systems or tissue chips (Figure 1B), are used to model tissues and organs by simulating physiological and pathological tissue components and arrangement, structural composition, and dynamic components (gas, blood, force, etc.) [2,10]. They employ advanced technologies such as microfabrication, microfluidics, and bioprinting to establish the corresponding structure and dynamic microenvironments on a tiny chip. In addition, OCs can be integrated with a sensor system to achieve continuous and automatic detection of biochemical and physical parameters [2,11,12].

In contrast to the various abovementioned detection and test platforms, OCs have unique properties in terms of tissue arrangement, biomechanical clues, and engineering design. Tissues on an OC platform can be orderly arranged in three dimensions (3D), allowing many types of cells to work together to reflect the physiological balance of cells as well as cells and tissues within a controlled design integration [11]. Specifically, OCs can also include biomechanical clues, such as the tensile and compressive forces of a lung tissue or the haemodynamic shear stress of a vascular tissue. Differences in the simulation of biomechanical forces can cause variations in tissue inflammation and drug absorption [2,13,14,15]. Creating OCs involves the reverse deduction to human cell system engineering, and the reverse engineering of body tissues and physiological systems is very complex. Consequently, OCs tend to simplify organ complexity by avoiding a comprehensive model. OCs present the main characteristics of human tissues by designing ideal structures; however, these designs can still provide relevant and helpful cues in the formation and development of certain diseases and, thereby, help to treat them [16,17,18,19].

This paper first outlines the nature of OCs and their significance. It then discusses their manufacturing-related materials and methodology, and highlights the applications of OCs in drug screening, disease modelling, and treatment. Finally, the future perspective of OCs is presented.

## 2. Manufacture of OCs

The manufacture of OCs involves a complex process that requires materials that are suitable for microfabrication with essential biocompatibility. When selecting materials for OCs, a confined long-term cell culture microenvironment should be considered, with multiple assays in sequence or synchronisation. Agreeable materials should simulate components to provide a safe and stable framework beneficial to cell growth and migration; they should also enable material exchange and signal communication between cells. These materials are referred to as ‘organ materials’ or ‘chip materials’ (Table 1). After the general structure of OCs is created with agreeable materials and advanced technology, the chips are subjected to specific environmental parameters, such as electromechanical stimuli and dynamic microenvironments. This process also can involve biosensing installation. The detecting elements are placed in the OCs, and the transduction components and signal-processing devices are connected to the outside.

### 2.1. Materials

The materials used in manufacturing and simulating biological components in OCs are referred to as ‘organ materials’. They are used mainly in the form of hydrogels and simulate the extracellular matrix of living cells and tissues.

Hydrogels can be of natural, synthetic, or hybrid origin. Natural hydrogels exhibit satisfactory biocompatibility with functional sites for cell adhesion and communication [20,21]. For example, collagen [22] and gelatin exhibit low immunogenicity and extensive structural domains for cell adhesion. They can create a cellular microenvironment similar to tissues [21,23]. As an example, by integrating collagen I hydrogel on polydimethylsiloxane (PDMS) gear, stem cell-derived endothelial cells grow on collagen membrane, and then, with collagenase, morphological changes in membranes and cells with progressive degradation of collagen can be studied [24]. Collagen–elastin (CE) membranes can also be cast onto the surface of PDMS moulds for better culturing of cells, as they have mechanical properties similar to those of in vivo biofilms [25]. Polysaccharides, chitosan, alginate, and hyaluronic acid can be modified in response to light, pH, temperature, and ion concentration. They are commonly used to encapsulate cells and for bioactive factor-controlled releasing [26]. In another study, chitosan was prepared as microspheres loaded with anticancer drugs, and drug release was controlled by two types of cross-linking: tripolyphosphate (TPP) and glutaraldehyde (GTA). Cumulative drug release was greater at lower pH values, demonstrating the pH-responsive nature of chitosan [27]. Alginate is considered an ideal substrate for the in vitro construction of muscle models, which can successfully induce ventricular myocytes and vascular smooth muscle cells to form striated and smooth muscle tissues by specific cues [28]. Natural polymers usually show weak mechanical properties, poorly controlled chemical and physical properties, and fast degradation rates [21,29]. Natural hydrogels can be chemically modified to cross-linkable methacrylate(MA), such as alginate (MAA) [30] and methacryloyl gelatine (GelMA) [31,32]. Synthetic polymers have high reproducibility in synthesis and biological experiments [20,33], such as cell cultures, with stable results [21]. These synthetic hydrogels can be prepared from polyethylene glycol (PEG), polylactic acid (PLA), poly (lactic-co-glycolic acid) (PLGA), polyvinyl alcohol (PVA), polyacrylamide (PAAM), poly (hydroxyethyl methacrylate) (PHEMA), and polyurethane (PU). As a material that mimics natural ECM, researchers have explored the effects of PAAM’s modifiable physical properties on the morphology, skeletal structure, and cell expression of kidney podocytes and have demonstrated them to be suitable for use in building kidney microarray models [34]. However, a significant disadvantage of synthetic polymers is that they lack cell-adhesion ligands and cytocompatibility [20]. Both natural and synthetic materials can be combined and integrated to take advantage of their complementary properties to form a rational design for on-demand physicochemical attributes, such as the combination of poly (ethylene glycol) diacrylate (PEGDA) with GelMA [35] and PEG with fibrinogen [36] for 3D printed scaffolds for cell seeding. Hydrogels can be coated on the surface of a chip channel. PDMS microfluidics have a gel as a cellular adhesive layer material to simulate the tissue environment in channel walls [37,38,39].

Cells can be of different types, including primary, immortalised, adult stem, embryonic stem cells, and human induced pluripotent stem cells. Among them, primary cells have a cellular phenotype most similar to that of human cells, but they have a limited lifespan and are difficult to obtain and preserve. Phenotypic differences exist between batches of primary cells [40]. Immortalised cells do not suffer from long-term preservation, and phenotypic differences and can be obtained with better reproducibility; however, they undergo genotypic and phenotypic drift modifications, exhibiting different functions from those of the original tissue or organ [41,42]. Stem cells, however, can differentiate into adult cells without phenotypic differences or concerns regarding long-term preservation. One commonly used adult stem cell is the mesenchymal stem cell, which supports differentiation into different types of cells; however, different methods of isolation and culture can likewise lead to phenotypic differences [43]. Embryonic stem cells are suitable for OCs, allowing for unlimited proliferation and differentiation, and are highly relevant to human cells; however, their use is ethically controversial [44]. Currently, human induced pluripotent stem cells are the most commonly used OCs because of their great relevance to humans, and they can be differentiated into human cells without ethical problems [43,45].

Biopsies are also frequently assembled in the design of OCs, and their use brings intercellular integration and overall function of the tissue within the OCs closer to that of living tissue [46,47]. For example, researchers grew mouse colon crypt biopsies on the chip, allowing and designing tissue blocks to proliferate and differentiate within the chip, with the final chip model exhibiting better cellular integration and a specific appearance of the structure [48]. Biopsied human skin tissue was used in a toxicity study in a multi-organs-on-a-chip (MOC) model for drug screening [19]. In addition, in building personalised disease models of patients, many microarray platforms are fitted with the disease site of the patient’s biopsy, such as the airway component of a patient with chronic obstructive pulmonary disease (COPD) [49].

However, the effect of cell sex on the results of experiments is often ignored when choosing cell types. Researchers often prefer male cells or tissues because female models are influenced by reproductive hormone levels, which makes results more unpredictable. Moreover, it is usually believed that sex differences have little impact on systems other than the reproductive system and are of little value to study [50]. Nevertheless, an increasing number of studies have revealed sex differences in non-reproductive tissues. For example, the brains of males and females show significant differences in anatomy and physiology, leading to sex differences in neurophysiology and behavior [51]. Males are more biased towards autism spectrum disorders [52], while females are biased towards depression and anxiety disorders [53]. In addition, there are sex-specific differences in the cardiovascular [54] and immune systems [55].

The materials used to create and simulate the non-biological components of OCs can be referred to as “chip materials” or structural materials of the chip, such as microfluidic devices and the barrier membranes between different types of cells. Elastomeric materials, such as PDMS [56,57,58], poly (octamethylene maleate (anhydride) citrate) (POMaC), thermoplastics, and inorganic materials are commonly used today.

PDMS is economical, low-cytotoxic, and easy to process. PDMS is transparent and, when assembled into a frame structure of an OC, can be viewed directly outside the chip for imaging, which is a major highlight of PDMS materials [59]. However, PDMS also has shortcomings while manufacturing OCs: PDMS is somewhat hydrophobic, preventing some cells from adhering to growth, and adsorbs small hydrophobic molecules, such as some of the drugs tested, which can affect the results of the experiment [60]. The uncross-linked part of the cured PDMS can leach into the solution [61]. PDMS is prone to solution evaporation, which significantly alters the volume, concentration, and equilibrium on a micro-scale, or even forms bubbles that can block gas–liquid flow or damage cells [56,61,62]. In addition to PDMS, another elastomeric material, POMaC, is used in the construction of vascular scaffolds, forming a network structure with many microchannels that mimic the scaffold structure of a blood vessel [63].

Thermoplastics can retain the visibility, biocompatibility, and chemical stability of PDMS materials while overcoming certain disadvantages of PDMS materials [64]. For example, cyclo-olefin polymer (COP) is transparent in the visible and near-UV regions, has low self-fluorescence for visibility, has no effect on hydrophobic drug distribution, and has ultra-low water vapour permeability, which facilitates cell culture while limiting sample evaporation [64,65]. Polymethyl methacrylate (PMMA) is rigid and transparent, and has very low fluorescence intensity, and is commonly used in the construction of OCs [46,66].

Among inorganic materials, glass is commonly used to prepare OCs. Glass is transparent, suitable for real-time imaging, and does not attract hydrophobic molecules [67]. However, owing to its impermeability, glass is unsuitable for cell culture in an enclosed environment [56].

**Table 1:** Summary of materials used for organs-on-chips.

Classification				Strengths	Weaknesses
**Organ material**	Hydrogel	Natural [68]	Collagen	Biocompatible [69]; Biodegradable [21]; Low immunogenicity; Extensive cell adhesive domains [21,23]; Suitable for cell growth and migration; Structure similar to ECM [26]	Weak mechanical properties [70]
			Gelatine		
			Chitosan		
			Alginate		
			Hyaluronic acid		
			Fibrin		
		Synthetic [20]	PEG, PLA, PLGA, PVA, PAAM, PHEMA, PU	Controllable mechanical properties; Stable in batch-to-batch; Controllable degradation properties; Chemical modification	Lack of cell adhesion ligands; Inadequate biocompatibility
		Hybrid	PEGDA/GelMA [35]	Appropriate mechanical properties; More bioactive sites	-
			PEG/fibrinogen [36]	PEG was functionalised to promote cell growth	-
	Cells and tissues	Primary cells [40]		The most phenotypically similar to cells in vivo	Extraction difficulty; Inconstant functionality; Short lifespan; Individual difference
		Immortalised cells [41,42]		Infinite survival; Retention of activity; Repeatable	Low phenotypically similar to cells in vivo
		Embryonic stem cells [44]		Pluripotent; Infinitely proliferative	Ethical restrictions
		Adult stem cells [43]		Easy to extract relatively	Limited differentiation ability
		Human induced pluripotent stem cells [43,45].		Retained human relevance; Great differentiation potential; Without ethical restrictions	Individual difference; Low reprogramming output; Genomic instability
		Biopsies [46,47]		More accurate information on the tissue [48]; Maintain the natural extracellular matrices and three-dimensional tissue structures [48]	Cannot survive more than 48 h in ex vivo culture mostly
**Chip material**	Elastomerics	PDMS [56,57]		Economic; Low cytotoxicity; Ease of processing; Transparent [59]	Hydrophobic; High ability to adsorb small hydrophobic molecules [61]; High gas permeability [56,61,62]
		POMaC [63]		Biodegradable; Biocompatible; Desired mechanical properties	
	Thermoplastics	COP, COC, PC, PS, PMMA		Economic; Transparent; Low absorption; Appropriate gas permeability [64,65]; Low auto-fluorescence [46]	
	Inorganic materials	Glass		Transparent; Stable physical and chemical properties [67]	Diseconomy in fabrication; High gas impermeability [56]

Annotations: PEG, polyethylene glycol; PLA, polylactic acid; PLGA, poly (lactic-co-glycolic acid); PVA, polyvinyl alcohol; PAAM, polyacrylamide; PHEMA, poly (hydroxyethyl methacrylate); PU, polyurethane; PEGDA, poly (ethylene glycol) diacrylate; GelMA, gelatine methacryloyl; PDMS, polydimethylsiloxane; POMaC, poly (octamethylene maleate (anhydride) citrate); COP, cyclo-olefin polymers; COC, cyclic olefin copolymer; PC, polycarbonate; PS, polystyrene; PMMA, polymethyl methacrylate.

### 2.2. Techniques and Environmental Parameters

**Figure 2:**
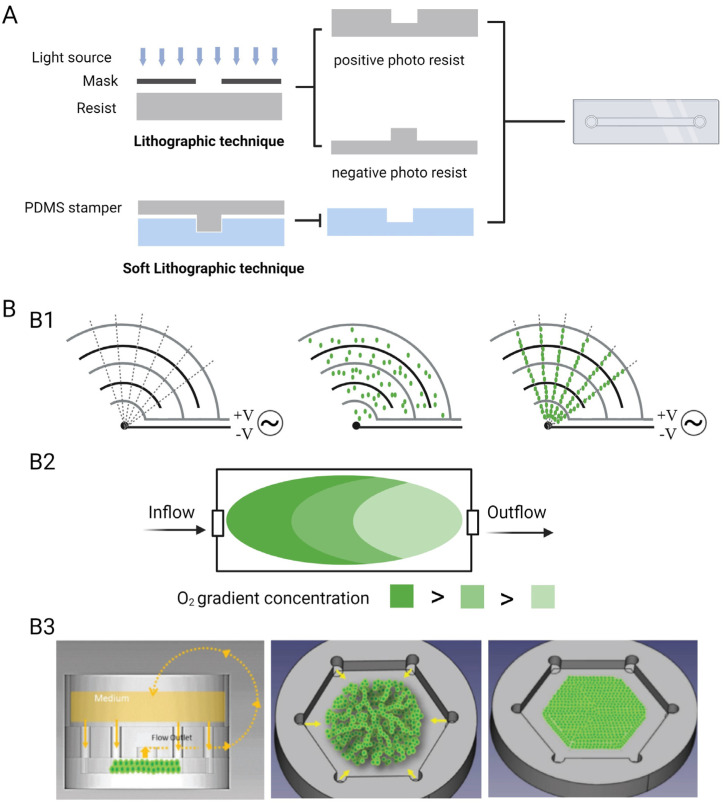
Manufacture of OCs. (**A**) Techniques used in OCs−photolithography technology and soft lithography technology in OCs. (**B**) Environmental parameters influence the tissue formation: (**B1**) The ac DEP voltage distributes liver cells in magnetic direction. (**B2**) Oxygen gradients create tissues with different metabolic levels. (**B3**) Liver cells form hepatic cord along the direction of radial flow. (Reprinted from ref. [93] with permission.)

Microfabrication technology is used in the fabrication of OC hardware templates or ECM scaffolds [71,72,73], using photolithography [74] and soft lithography technologies. Photolithography technology focuses on the formation of UV-sensitive materials, where the constructed hardware can be directly applied to OCs or used as a master for soft lithography. Soft lithography technology is a technique for infusing materials such as PDMS into a master plate and removing the template after shaping to obtain a soft and flexible microstructure (Figure 2A). Multi-layered soft lithography utilises 3D stamping techniques for the tight assembly of multi-layered structures [71]. 3D printing enables stereoscopic structure efficiently with high fidelity and wide dimensional pattern ranging from micro- to macroscale without templates or masks [75,76]. The cell and tissue components are mainly printed in 3D, using bioprinting technology to create tissue structures [77,78] or using electrospinning technology to first form a cell growth scaffold [79] and then grow the cells to promote cell growth and distribution.

Microfluidics is a core technology in OC fabrication, which controls the flow of fluid in channels of less than 1 mm, allowing for better simulation of the dynamic cellular and tissue environment [80]. In microchannels, the liquid flows directionally and steadily, without turbulence, and can be used to maintain the stability of chemical gradients over long periods of time [81]. Microfluidics is used to control dynamic changes, such as the flow of the culture fluid delivery and the exchange of gases, simulating the dynamic circulatory changes in the human body’s ministries [2,26,73,80,82].

Of course, owing to the flexibility of OC research, various technical supports are required for target OCs under different research objectives, such as the establishment of a liver sinusoidal model with the help of bi-directional electrophoresis techniques and the design of electrodes that enable the radiation electric field to form the hepatocytes into a hexagonal arrangement [83] (Figure 2(B1)).

To better simulate the tissues and organs, designers have replicated many environmental parameters of the body in terms of the microenvironment. In terms of structure, specific tissue morphology and function require different ECM structures for maintenance [84], e.g., the survival of hematopoietic stem cells (HSCs) is closely linked to the bone marrow ecological niche [85]. In terms of biochemical stimulation, specific cytokines and chemokines are delivered to tissues and cells to promote cell survival, proliferation, and migration [86], such as the addition of MCP-1 to GelMA to induce monocyte chemotaxis [87]. In tissue homeostasis, microfluidics provides dynamic nutrients and remove metabolic waste to maintain intracellular homeostasis and control the oxygen gradient between different tissues [88,89] (Figure 2(B2)). In terms of mechanical stimulation, electrical stimulation largely affects cellular morphology and activity, and appropriate electrical stimulation can improve synaptic extension in nerve cells [90] and the assembly of specific morphology in muscle cells [91]. Additionally, appropriate mechanical stimulation can similarly affect cellular alignment and growth trends, such as fluid shear stress [92,93] (Figure 2(B3)).

### 2.3. Sensors

An important piece of combined equipment for OCs is the sensors. As OCs continue to evolve, developing integrated sensors is vital for monitoring the microenvironment within the chip in real time, continuously and precisely. Sensors can be divided into physical sensors that display pH, temperature, and oxygen, and biosensors that display multiple biomarkers of metabolic processes [94], which can be further divided into optical and electrochemical sensors based on different principles [10].

Optical sensors use light at specific wavelengths to directly detect the absorbance and fluorescence intensity of a substance or indirectly to determine the surface plasmon resonance (SPR) on the probe surface and the binding of the analyte to the probe to determine the analyte concentration [10]. Optical sensors can detect a limited range of substance concentrations; for example, when detecting pH, they require the addition of absorbable phenol red for colour development and, only in the pH range of 6.5–8.0, linearity reflects well and data are accurate [95,96]. However, optical sensors are advantageous in low-oxygen environments because their measurement data are independent of redox reactions [97,98].

Electrochemical sensors are more indirect and flexible, which can be used for more precise quantitative studies. They consist of at least three electrodes for countering, referring, and working. The working electrode has a conductive gel or noble metal containing the reactant (oxidase) attached to it, which reacts with the analyte to produce an exchange of anions and cations. Then, the analyte concentration is calculated by detecting the amperage, potential, and resistance between the working and counter electrodes [10,96]. Electrochemical sensors are designed to be more flexible and to facilitate real-time continuous monitoring [99,100]. Some researchers have used conductive PEDOT:PSS hydrogels wrapped with glucose oxidase (Gox) to prepare working electrodes to create miniature, non-invasive, portable glucose sensors [101]. Researchers have also developed an intestinal barrier chip model to measure transcutaneous resistance within transwell chambers to assess barrier integrity [102,103].

Finally, the construction of an ideal sensor should rely on a combination of optical and electrochemical sensors on demand. In a MOC model, researchers designed an automated monitoring platform with physical sensors and electrochemical biosensors connected in microfluidic channels to reflect micro-environmental parameters (potential of hydrogen, oxygen, and temperature) and biomarkers within the model, respectively. The entire device was placed directly under a microscope to observe the internal morphology of the MOC. MOC also enables in situ and real-time monitoring of the microenvironment, biological components, drug screening, and internal morphology [95].

### 2.4. Cell Culture Medium

When a single OC, involving only a single cell type, is generated, a culture medium initially formulated for conventional cultures can be used. However, when generating single/multiple OCs involving multiple cell types, the choice of medium becomes more complex because each cell type has specific nutrients and growth factors to sustain growth and function [104]. Therefore, one of the most critical challenges for multi-organ chips is to be able to provide different tissues on the system with a blood-like universal medium to meet the nutritional needs of different cells. So far, there are two ways to solve this problem. One is to improve the properties of the culture medium to expand its application range, and the other way is to utilize the endothelial cell barrier for nutrient separation [16]. For instance, in the connected liver–kidney system, mixing liver-specific medium and kidney-specific medium in a 1:1 manner can come close to meeting the growth needs of both types of cells [105]. In addition, there are multicellular chips that simulate adipose tissue [106] and a multi-organ chip model of liver–fat–skin–lung interaction [107]. However, with an increasing number of organ types in the system, this multiple-mixing approach may result in a less effective medium, as each organ or tissue ends up with a sub-optimal medium, which can greatly affect the functionality and physiological relevance of the system. The connected system can be designed as a single channel or recirculation system, so that the medium can be replenished or replaced at any time, and the barrier formed by the vascular endothelium can also be utilized on the chip platform to segregate the different types of cells, so that they can be cultured with their own optimal medium.

## 3. Applications

OCs are popular and widely used, including applications in drug absorption, distribution, metabolism, and excretion; in modelling diseases; in building medical resources; and in individualised drug administration. The following is a brief description of the design of OCs for use in these areas.

### 3.1. Drug Screening

**Figure 3:**
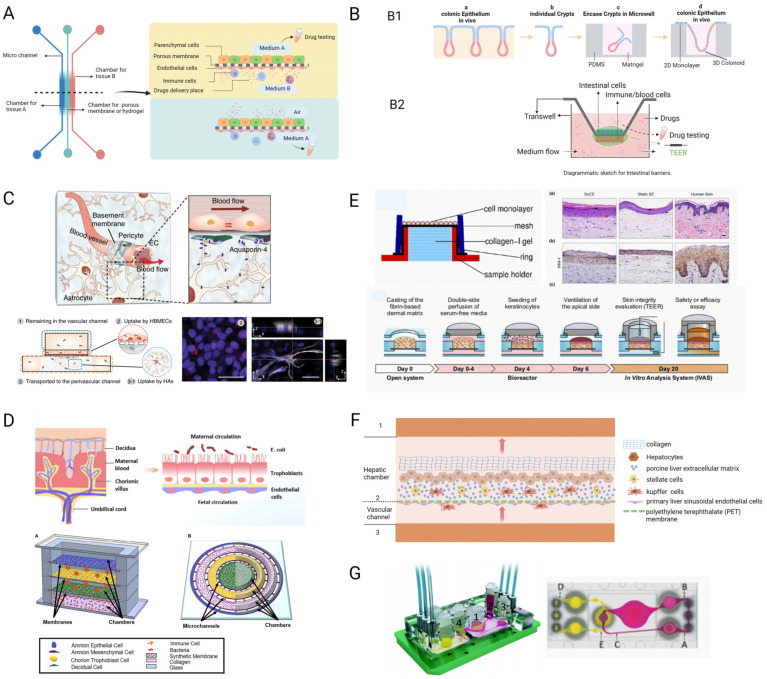
Drug screening by OCs. (**A**) Diagrammatic sketch for barriers. Intestinal barrier, blood–brain barrier, and placental barrier in yellow, airway barrier and skin barrier in green. (**B**–**G**) The designs of intestinal barrier chip model (**B**), in which B1 is primary colonic crypts-on-a-chip model and B2 is a Transwell modle for intestinal barrier-on-a-chip model. Blood–brain barrier chip model (reprinted from ref. [18] with permission) (**C**), mMaternal–foetal barrier chip model (reprinted from refs. [17,116] with permission) (**D**), in which A was designed to create an infectious preterm birth model to study fetal membranes and B was designed to mimic the feto-maternal interface, including the fetal membranes and maternal decidua. Skin-on-a-chip model [116,121,122] (reprinted from refs. [121,122] with permission) (**E**), Liver sinusoidal chip model (**F**) and ADME MOC model (reprinted from ref. [19] with permission) (**G**), in which pink represents blood flow circuit, yellow represents an excretory flow circuit, numbers represent the four tissue culture compartments for intestine (1), liver (2), skin (3), and kidney (4) tissue. (A, B, C) represent three measuring spots in the surrogate blood circuit and (D, E) represent two spots in the excretory circuit.

Applications of the intestinal barrier chip model, liver sinusoidal chip model, blood–brain barrier chip model, maternal–foetal barrier chip model, skin-on-a-chip model, and ADME MOC model for drug detection are introduced. The diagrammatic sketch for barriers is shown in Figure 3A.

#### 3.1.1. Intestinal Barrier Chip Model

The intestinal barrier is an important barrier to the absorption of most orally administered drugs, and numerous localised chip models of the intestine have been designed to explore the direction of model design at the end-organ level, such as the colonic crypts-on-a-chip model [48] (Figure 3(B1)). Primary crypts isolated from mice were mixed and cultured in 2D and 3D patterns on a microstructure consisting of PDMS micropores and matrix gel micro pockets to produce continuous millimetre-scale colonic epithelial tissue. This is an effective exploration of the colon-on-a-chip model. It is novel and ingenious to choose targeted tissues and combine 2D and 3D culture methods to better simulate the tissue composition and structure of the crypt.

In contrast, the design of the intestinal barrier-on-a-chip model selected several major cells and chose a Transwell model for its structure (Figure 3(B2)). Caco2-BBE cells and HT29-MTX cells were cultured at a ratio of 9:1 in a mixture of 0.4 µm Transwell chambers to form an intestinal monolayer epithelium, and the basal side of the Transwell was planted with dendritic cells, forming the basic intestinal barrier model [103]. The use of the Transwell chamber was impressive, as it can be used to study cell migration and can also be applied to research the absorption and passage of drugs microscopically; it is the perfect hardware for the intestinal barrier model. In addition, researchers have addressed the problem of quantifying barrier function by measuring the transepithelial electrical resistance (TEER) in the intestinal epithelium. Dendritic cells and HT29-MTX cells respond to exposure to drug components or inflammatory mediators in the culture medium, increasing transmembrane resistance. The absorption of drug breakdown products or the secretion of mucin into vesicles increases transmembrane electrical resistance. The higher the transmembrane resistance, the better the barrier function [16]. Researchers also modified the human Caco2 intestine chip by integrating a micro-oxygen sensor into an in situ oxygen measurement device and placing the chip in an engineered anaerobic chamber to create a physiologically relevant oxygen gradient between the human intestinal epithelium and microvascular endothelial cells, which are cultured in parallel channels separated by a porous matrix-coated membrane within the device. Modified intestinal chip allows stable co-culture of highly complex anaerobic and aerobic intestinal bacterial communities in the same channel as mucus-producing human villous intestinal epithelium, while simultaneously monitoring oxygen levels and intestinal barrier function for at least 5 days in vitro [108].

#### 3.1.2. Blood–Brain Barrier Chip Model

Another barrier to drug delivery is the blood–brain barrier (BBB), which is an important threat to the healing efficacy of nervous system drugs. The BBB is the boundary between the central nervous system and circulatory system that controls transportation between the blood and the brain, limiting the penetration of drugs. It consists mainly of the outer vascular endothelium, middle pericytes, and inner network of astrocytes. The ends of these astrocytes cross pericytes to come into contact with the vasculature and control the inflow of water through aqueous channel 4 (AQP4) [109,110].

Based on this structural basis, researchers produced a simplified BBB-on-a-chip model [111]. The basic structure of the micro-platform was built using PDMS and consisted of an upper layer, polycarbonate porous membrane, lower layer, and a slide (Figure 3C). The upper layer was seeded with 2D human brain microvascular endothelial cells (HBMECs), and the lower layer was placed with a network of pericytes and 3D astrocytes, between which a porous membrane was placed, significantly reducing the distance between them [18]. Primary cells were selected so that the final microarray replicated the specific markers, membrane transporters, and receptors associated with the BBB to a greater extent.

One of the main mechanisms by which natural HDL is known to pass through the BBB is class B scavenger receptor 1 (SR-B1) [112,113]. Researchers designed mimetic HDL nanoparticles with apolipoprotein A1 (eHNP-A1) and validated this mechanism using a BBB microarray model [18]. The nanoparticle solution was added to the upper medium and compared before and after blocking the SR-B1 channels. The amount of eHNP-A1 in the upper layer, that is, the vascular channel, increased significantly after blocking the SR-B1 channel, whereas there was no significant increase in eHNP-A1 across the semi-permeable membrane nor upon reaching the lower layer, that is, the microenvironmental layer in the brain. In addition, the researchers captured the location of eHNP-A1, demonstrated the distribution of 3D nanoparticles in the microarray model, and elaborated on the different cellular uptakes and receptor-mediated cytokinesis in this model (Figure 3C). The mechanism of HDL penetration of the barrier and the usability of the BBB-on-a-chip model validate each other.

#### 3.1.3. Maternal–Foetal Barrier Chip Model

The placental barrier is also known as the interface between the mother and foetus. This barrier consists of an external trophoblastic layer and an internal foetal endothelial layer. This barrier counts the circulation and communication between the mother and the foetus. The placental barrier-on-a-chip model has provided an excellent tool for studies related to drug and toxicity delivery between the mother and foetus; however, it surpasses this to include breakthroughs in preterm birth induction, hormonal regulation, and maternal–foetal communication. The toxic effects of drugs on the foetus are a major concern when medically administering drugs to pregnant women; however, many in vitro 2D and 3D assay platforms limitedly represent the complex conditions between mother and foetus, and the animal models differ significantly from humans in terms of gestational physiological structure and uterine environment. An excellent placental barrier-on-a-chip model should be created and should be taken a step further for researching the purposeful delivery of drugs from the mother to the foetus.

Researchers used PDMS material to create two microchannels separated by a porous polycarbonate membrane seeded with trophoblastic epithelial cells (BeWo epithelial layer) and foetal vascular endothelial cells (HUVEC layer) to mimic the maternal and foetal environments on either side of the barrier [17,114] (Figure 3D). The researchers verified the gestational deep vein. The inability of heparin, a therapeutic agent for thrombosis and embolism, to penetrate the placental barrier-on-a-chip model is consistent with previous findings. In addition, researchers tested the delivery of glibenclamide, a drug commonly used in gestational diabetes, in this model. The placental barrier microarray model demonstrated the inability of glibenclamide to enter the foetal side under the protection of breast cancer resistance protein (BCRP). Conversely, massive glibenclamide enters the foetal side under the action of BCRP inhibitors [114], validating the physiological protection of BCRP on the foetus. This shows that the placental barrier-on-a-chip model can reconstitute the transport function of the placental barrier and has great potential as a drug screening platform [115].

The above mentioned is a simpler model of the placental barrier, which lacks many important components to reconstruct the maternal–foetal interface [116], such as the lack of many important cellular components, e.g., amnion mesenchymal cells(AMCs) and chorion trophoblast cells(CMCs/CTs) [117]. There are two more well-established placental barrier chip models: one consisting of four parallel chambers culturing amnion epithelial cells (AECs), trophoblast cells, metaphase, and bacteria [118], and the other consisting of four concentric circular channels containing primary AECs, AMCs, CMCs/CTs, and metaphase cells [117]; the latter is superior, in that the four chambers are connected by fine ducts filled with extracellular matrix, which is more realistic than a simple PET membrane (Figure 3D). These designs are more similar in structure and composition to the placental barrier, and, with such models as a cornerstone, future studies on bacterial infections, drug therapy, etc., will be more accessible.

#### 3.1.4. Skin-on-a-Chip Model

The skin barrier is an obstacle that must be examined for all transdermal drugs, such as creams, ointments, solutions, and skin patches, commonly in the form of antibiotic cream for the skin, proprietary Chinese medicine suspensions [119], and pain relief patches for the nervous system [120]. Transdermal drug delivery has many advantages over traditional routes of administration, such as non-invasive administration and avoidance of the first-pass effect of the drug on the digestive system. The drug can act locally or systemically without significant side effects and has a higher safety profile. Animal models are often used in drug experiments to study transdermal drug delivery, but the species variability between animals and humans makes the results lack validity. With increasing restrictions on the use of animals in various countries, we advocate non-animal research methods. The use of skin equivalents is increasingly required for studies of drug penetration, skin irritation, and skin phototoxicity.

Researchers have developed a skin-on-a-chip model that mimics the structure of a Franz-diffusion cell by growing human keratin-forming cells (HaCaT) on a modified electrostatic spun membrane in the middle. The upper layer holds the sample fluid, and the lower layer is filled with type I collagen and connected to a microfluidic channel for easy extraction of the culture fluid and measurement of drug concentration. The lower layer can be removed for tissue staining. This model has been used to study the transdermal transport of caffeine. The maximum concentration of caffeine in the collected cultures was reached at the fifth hour, showing transport kinetics similar to those of human skin samples [121] (Figure 3E). In a full-thickness skin-on-a-chip model, a fibrin-based dermal matrix was used to construct a dermal scaffold and fill the upper surface and interior with keratin-forming cells and fibroblasts, respectively, resulting in a simple full-skin equivalent, which was also shown to be well structured by tissue staining [122] (Figure 3E). Biopsied skin tissue has also been used to replace monolayers of keratin-forming cells [123,124], to establish better models of full-thickness skin-on-a-chip. Other researchers have applied periodic mechanical stimulation to the skin based on a full-thickness skin-on-a-chip model to simulate circadian rhythms, resulting in an aging skin model that can be used for drug development and disease modelling in aging skin [125]. In addition, the subcutaneous vascular component was considered, and skin microarray models containing different vascular channels were created to detect vasodilatation and immune responses to skin stimulation [126,127]. These models are simple and convenient, but they contain only a small number of types of skin cells and lack some important skin accessories, such as nerves and hair follicles, where the hair follicle structure also has an impact on the absorption behaviour of the drug. There are aspects that have not yet been integrated into the skin-on-a-chip model, which is an area where breakthroughs are needed [128].

#### 3.1.5. Liver Sinusoidal Chip Model

The liver is an important site for drug metabolism, and hepatotoxicity is an unavoidable safety indicator in the drug screening process. The liver is the main organ for testing the effectiveness and safety of drugs. The basic liver functional unit is the hepatic sinusoid between the hepatic portal and central vein, consisting of an inner layer of porous endothelial cells, middle layer of hepatic stellate cells, and outer layer of hepatic parenchymal cells. Kupffer cells are also present inside the sinusoid, mediating communication and immune responses [88]. In particular, the partial pressure of oxygen in the hepatic sinusoids gradually decreases during the flow of blood from the periportal to the central vein to regulate the compartment and function of the liver [129].

Accordingly, researchers designed and improved the hepatic sinusoidal-on-a-chip model using glass material instead of PDMS material to create a hollow channel structure that avoids the hydrophobic and oxygen-permeable nature of PDMS. Primary LSECs and stellate cells are grown in the lower layer to form the vascular channels and intrahepatic environment, and primary human hepatocytes and collagen layers are grown in the upper layer, distributing the Kupffer cells within the lower two layers. Primary cells were selected so that the final model contained many specific proteins. In addition, researchers formed different oxygen partitions (oxygen-rich, intermediate, and oxygen-poor) by regulating the flow within the liver and vascular channels through engineering modelling and verified the oxygen partitioning by imaging the ratio of oxygen-sensitive and-insensitive fluorescent beads to form different metabolic gradients [16,88,89] (Figure 3F). The model validity was verified by a bile efflux test and an endothelial cell penetration test, using various imaging modalities. This model was also compared to a PDMS liver sinusoidal-on-a-chip model [130], in which three drugs (Nefazodone, Terfenadine and Acetaminophen) were dissolved and diluted into the vascular channels of the chip, and the effluent was collected daily to determine the remaining drug concentration and calculate the recovery rate. The recovery of acetaminophen, a hydrophilic drug, was 100% in both devices, whereas the recovery of Nefazodone and Terfenadine, hydrophobic drugs, was much greater in the glass device than in the PDMS device. All recoveries were 100% after the addition of drug-loaded LDL. These data demonstrate the drawback of the PDMS material in the fabrication of a chip device, namely, its ability to adsorb hydrophobic molecules [2].

#### 3.1.6. An ADME MOC Model

The MOC model, which is formed by multiple OC, is a more suitable approach for drug development, where drugs undergo absorption, distribution, metabolism, and excretion (ADME) processes in vivo, mainly involving the intestine, liver, kidneys, and organs of action of the target drug, such as the skin, brain, and even bone marrow [16,131,132,133,134,135].

Researchers designed an ADME-on-a-chip model by integrating the gut, liver, kidney, and skin using PDMS and PET membranes to prepare a bilayer of interconnected chambers, with the upper layer serving as the blood circuit and the lower layer as the excretory circuit, with the two layers connected by a proximal tubular culture chamber in the middle of the kidney (Figure 3G). The blood circuit includes an intestinal barrier culture chamber consisting of intestinal epithelium, a liver equivalents culture chamber consisting of liver parenchymal cells and stellate cells, and a skin culture chamber for biopsies and connected microchannels. The excretory circuit consists of a proximal tubular culture chamber and microchannels in the kidney [16,19]. This continuous MOC model was tested for LDH and glucose distribution, and there was a corresponding gap in LDH activity extracted in the different chambers, further suggesting that the four barriers play their respective roles in the passage of LDH [19]. The MOC model can be flexibly designed and adapted for the purpose of the study; for example, heart–liver–lung chip models to study drug effects on each organ [136], liver–kidney chip models, and liver–skin chip models to study drug toxicity [135,137,138,139].

With the need for constant sampling and testing during experiments, the establishment of a long-term, real-time, continuous testing system is unavoidable. A sensor system for in situ continuous monitoring has been established [95], which is a significant advancement in the development of MOC. MOCs are a flexible and beneficial tool in the drug development process, but their design flexibility also leads to a lack of widely accepted standards for the test tool, which is partly responsible for its limited popularity. Another challenge is the limited survival time of MOCs, which is difficult to match with the time required for drug ADME.

### 3.2. Disease Modelling

The use of OCs models of disease can provide data that are more in accordance with human physiological responses, allowing scholars to gain a deeper understanding of disease characteristics and trends. Their use also reduces animal consumption, as recommended by the “3Rs” principle (replacement, reduction, refinement) in animal ethics. Here, we focus on an airway-on-a-chip model, simulating disease models for influenza A and COVID-19 infection and using disease models to predict effective drugs for COVID-19 infection [140]; tumour-on-a-chip models, which predict tumour metastasis [141]; and a breast cancer–heart-on-a-chip model to detect chemotherapy toxicity in the heart [142].

#### 3.2.1. Airway-on-a-Chip Model

**Figure 4:**
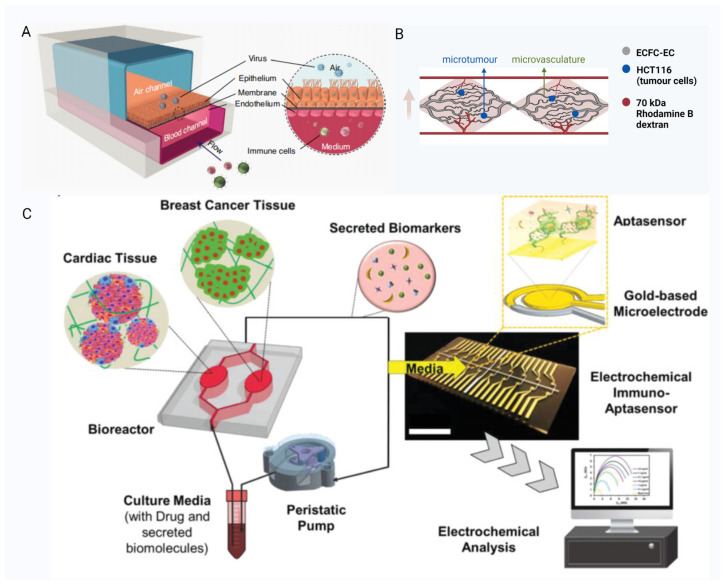
Disease modelling by OCs. (**A**–**C**) Diagrammatic sketches of an airway-on-a-chip (reprinted from ref. [140] with permission) (**A**), a vascular tumour-on-a-chip (**B**), and a breast cancer–heart-on-a-chip (reprinted from ref. [142] with permission) (**C**).

Researchers grew human bronchial airway epithelial cells and artery endothelial cells in a dual-channel microstructure created by PDMS and PET porous membranes to form an airway-on-a-chip model, combining air and blood channels [15,140] (Figure 4A). This model was infected with influenza A virus, and the infection model was verified by immunofluorescence staining, followed by treatment of the microarray model with an effective therapeutic agent for influenza A, which ultimately showed the effectiveness of the treatment; thus, this proved in reverse that the airway microarray model of viral infection was successful. The researchers then used this chip model to develop a COVID-19 infection airway-on-a-chip model of simulated novel coronavirus infection by pseudotyped syndrome coronavirus type 2 (SARS-CoV-2) and used it to predict effective drugs for novel coronavirus pneumonia in vitro. The predicted drugs were also effective in the prevention and treatment of infection in animal models of COVID-19 infection [140]. The use of this model has demonstrated that OCs models have enormous potential in the treatment of pandemic diseases, alleviating the urgency and burden to develop effective drugs rapidly on occurrence of serious pandemics.

#### 3.2.2. Tumour-on-a-Chip Models

Researchers have developed vascularised micro-tumour-on-a-chip models for screening effective chemotherapeutic agents [16,141,143,144]. The model was built in PDMS material and bottomless 96-well plates using human vascular endothelial cells in a diamond-shaped culture chamber to self-organise into a 3D microvascular model, growing colorectal tumour cells that differentiated and formed micro-tumours (Figure 4B). This model allows for clear localisation of cell distribution and interactions by histochemical staining. Interestingly, many clinically used chemotherapeutic drugs could be effective in this 3D micro-tumour-on-a-chip model, whereas some of them were not effective when the drugs were also applied to 2D tumour tissue [141]. These findings highlight the superiority of OC models for drug screening compared with 2D cell cultures. It is also possible to build OC models of other tumours to select effective chemotherapeutic drugs [142,145,146].

In addition, many cancer metastases-on-chips have been established to clarify the mechanisms of metastasis in different tumours [147]. Establishing a personalised multi-organ microarray model of a tumour patient can help predict the metastatic activity and trend in that patient’s tumour in advance and is a potential area for future study and applications.

#### 3.2.3. Breast Cancer–Heart-on-a-Chip Model

Chemotherapy-induced cardiotoxicity (CIC) is the most likely adverse event in the course of chemotherapy for oncological diseases and is unpredictable, unless irreversible heart failure occurs. Based on clinical experience, researchers chose breast cancer for cardiotoxicity and established the cardiac–breast cancer-on-a-chip model [142]. The researchers co-cultured cardiomyocytes, fibroblasts, and myofibroblasts differentiated from human iPSCs in GelMA hydrogels to form mock heart spheres and similarly cultured breast cancer cells in GelMA, placing them separately in two linked microculture chambers. Addition of TGFβ1 to induce cardiac fibrosis was used to investigate whether a certain degree of myocardial fibrosis promoted the development of CIC (Figure 4C). The chip platform also incorporated an electrochemical multi-array sensor for real-time, continuous detection of multiple biomarkers such as CK-MB, cTnT, and HER-2 [148,149]. Treatment with doxorubicin (DOX) nanoparticles resulted in changes in biomarker indicators, reflecting the suitability of the platform. This microarray model is more of a conceptual model for predicting CIC, but with the concept proposed, a truly practical model is being prepared, and replacing all the cell sources in this model with patient-specific sources may be the way to proceed.

### 3.3. Treatment

In addition to drug detection and disease modelling, OCs have great potential for use as a flexible medical resource, a development that represents an unexpected breakthrough for both medical treatment and OC itself. Perennial bone marrow-on-a-chip [150], vascular-on-a-chip as a therapeutic scaffold [63], and foreign body corresponding-on-a-chip [87] as an aid for treatment monitoring are currently being investigated.

#### 3.3.1. Bone Marrow-on-a-Chip

The use of OCs as a tool for cultivating resources for disease treatment is a breakthrough in the application of various biomaterials. All types of blood cells originating from HSPCs and their survival are closely linked to the bone marrow cell ecotone, but modelling all aspects of the bone marrow cell ecotone in a more comprehensive way remains unaddressed The bone marrow cell ecotone is structurally complex and consists mainly of cells, such as bone marrow stromal cells and MSCs; it includes a variety of signalling molecules and extracellular matrix components [85,151,152,153].

**Figure 5:**
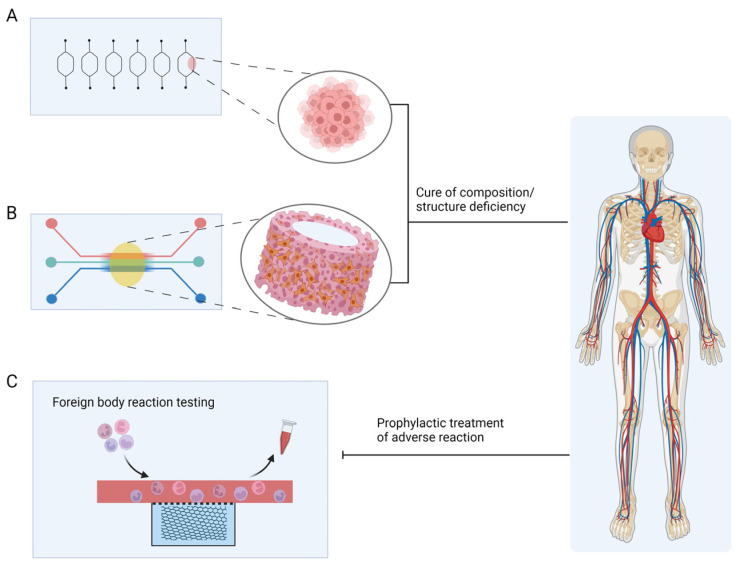
Treatment by OCs. (**A**–**C**) The treatment concept maps of bone marrow-on-a-chip [150] (**A**), vascular-on-a-chip (**B**), and foreign body corresponding-on-a-chip (**C**).

The researchers learned from previous superior HSPC culture methods and used 3D ceramic scaffolds of hydroxyapatite-coated zirconium oxide [154], which mimics the porosity and strength of osteochondral stroma; it is filled with MSCs and HSPCs, in which MSCs produce signal molecules and ECM components. It can form a bone marrow model after one week of incubation in vitro. The bone marrow model was placed on a PDMS microchip platform consisting of two independent circular channels [139,150]. In this model, HSPCs remained in their original state after four weeks, and the ecotone composition remained unchanged, similar to the natural bone marrow ecotone [150]. Establishing this model has helped expand the sources of HSPCs and exploit the potential of an in vitro blood bank of HSPCs that can be used for the treatment of related haematological disorders, which has made them independent of bone marrow donation. This concept was developed to provide inspiration for the treatment of many other systemic diseases (Figure 5A).

#### 3.3.2. AngioChip

Some researchers have used OCs to breed a vascular scaffold chip (AngioChip), which is more biocompatible and promotes vascular regeneration (Figure 5B). The POMaC solution was injected into a PDMS porous mould designed by AutoCAD, it was UV-irradiated to form glue, and the PDMS mould was removed to obtain the scaffold structure, which made the final scaffold chip flexible. The scaffold structure was coated with gelatine and placed in a microfluidic PDMS material chip device. HUVECs were infused into the scaffold via a microfluidic system and left to stand until the cells adhered to the scaffold to form a 3D HUVECs vascular scaffold structure [63]. A rat vascular scaffold chip made in the same way was used in Lewis rats in either the artery bypass configuration or the artery-to-vein configuration, which established good blood perfusion and demonstrated excellent biocompatibility. One week after implantation, new angiogenesis was found around the scaffold, which was maintained in vivo for at least five weeks [63,155]. Depending on the requirements, cellular components of different compositions can be infused into the scaffold: HESC-derived hepatocytes with HMSCs for liver-vascular-on-a-chip and HESC-derived cardiomyocytes with HMSCs for heart-vascular-on-a-chip [155]. AngioChip grown using the OC is stable and can be directly anastomosed, allowing analytical exchange inside and outside the vessel and contributing to extensive tissue remodelling. Cells of patient origin can be selected to make the AngioChip a better fit for the patient and better for therapeutic use, but more complete and industrial methods of making the AngioChip are required before this can be performed.

#### 3.3.3. Foreign Body Corresponding-on-a-Chip

In addition to using OCs to cultivate therapeutic resources, researchers have created a monitoring and prevention platform that can detect foreign body responses in vivo [87]. The proposed design further extends the scope of application of OCs platforms. Implantable devices and biomaterials are already widely used in the treatment of diseases, but most implants have an immune cell foreign body response in the host body, which eventually leads to treatment failure or even more serious systemic accidents [156,157,158,159].

The foreign body response-on-a-chip is multi-layered and made of PDMS material, consisting of a foreign body (tiny titanium beads wrapped in GelMA) culture chamber at the bottom, a PET semi-permeable membrane in the middle, and a vascular channel containing HUVECs and monocytes in the upper layer [87,95,160]. GelMA gels in the foreign body culture chamber contain MCP-1 factors that mimic the chemokines released by cells exposed to foreign bodies and attract monocytes. Immunofluorescence staining demonstrated the dynamic approach of monocytes to titanium beads under microfluidic conditions and, ultimately, revealed that human monocytes from different donors responded to titanium beads at different levels in this microarray platform and that the phenotype of monocytes near titanium beads does not consistently show M1 or M2, which is consistent with individual variability in immune responses [87]. This suggests that, prior to implantation treatment, time-permitting circulating mononuclear cells from the patient can be extracted for implant-specific immune response assays, which can, to some extent, predict trends in immune levels in that patient post-implantation and can guide prophylactic treatment (Figure 5C).

## 4. Future Perspectives in Precious Medicine and Wound Healing

### 4.1. Precision Medicine

Another breakthrough in OCs is individualised medicine, where tissue from clinical patients is used to create OCs, which is similar to the application of disease modelling, but the two are incomparable. Patient-personalised disease models are used to determine disease progression, applicable drugs, and treatments [161,162]. Precision medicine is a thorny path that has been yearned for by the medical industry in recent decades; however, it is still struggling. Perhaps the application of OCs in this area can lead to new concepts and breakthroughs.

Currently, OC is commonly used in precision medicine for tumour drug screening, where patient-derived tumour cells, tissues, or pathological sections are cultured in a microchip, and effective drugs are screened in vitro to guide the patient’s clinical treatment plan [163]. It is personalised and has profound implications for subsequent treatment of patients. Although cells cultured in vitro cannot mimic the all-sided composition and characteristics of tumour tissue in vivo, the selection of appropriate tumour sections for microtissue culture allows for long-term and comprehensive drug detection [163,164].

**Figure 6:**
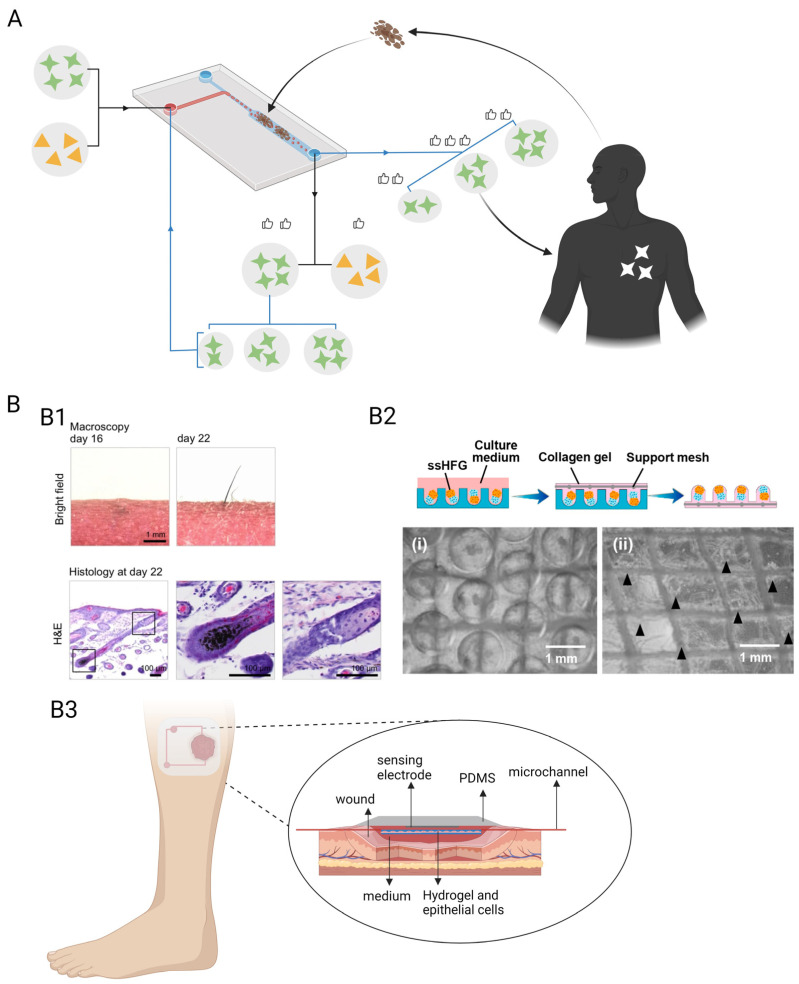
Future perspectives. (**A**) OCs help select the drug types and dosage precisely. The yellow and green shapes represent two different drugs. (**B**) Hair follicle tissue formed in vitro (reprinted from ref. [178] with permission) (**B1**). The boxed areas in the left panels are shown at a higher magnification in the right panels. Hair follicle tissue cultured in mass production in PDMS microwell array (reprinted from ref. [179] with permission) (**B2**). Representative photographs depict HFG appearance prior to (**i**) and after (**ii**) the collagen gel was removed from the HFG chip. Arrowheads indicate HFGs. And an assumption for OCs repairing skin in vivo (**B3**).

Researchers used tumour tissue from two mesothelioma patients, cultured them in vitro in OCs, and then used the same two chemotherapy combinations for each chip: carboplatin/pemetrexed and cisplatin/pemetrexed. They found that tumour tissues from both patients showed different results for the two chemotherapy regimens. Genomic testing identified one of the mutated loci in patient #1 and no mutation in patient #2 [165]. This study demonstrated the role of OCs in precision medicine for tumours. In addition to the precise selection of drug types, OCs are useful for determining precise drug dosage (Figure 6A). In two patient-derived tumour tissue chip models, the investigators administered each treatment regimen and found that #2 was not dose-dependent for cisplatin; therefore, dosage of cisplatin was appropriately reduced during treatment to mitigate unnecessary toxic effects [165]. Researchers established an OC model containing mouse brain tissue sections and infused the microarray with different doses of STS-simulating chemotherapy drugs, ranging from 10 nM to 6 μM. Tissue staining revealed that the number of apoptotic cells increased with increasing STS concentrations [123]. This suggests that OCs have the potential to test precise drug dosing; however, many factors not included in the microarray model, such as the ratio of drug uptake and metabolism, need to be considered in the process of clinical translation.

In addition, the application of organ chips in precision medicine includes accurate predictions for timely prevention and treatment. For example, many current OCs built using patient tumour tissue predict metastatic trends in tumours [147] or study the toxic effects of chemotherapeutic drugs on other organs. Except for tumour diseases, the foreign body response chip model mentioned above is also a good example of accurate prediction.

OC in precision medicine is widely applicable and not limited to oncological diseases, such as the establishment of airway-on-a-chip for COPD [49], vascular perfusion-on-a-chip [166], and intestinal microbiome-on-a-chip [167,168].

Personalised OC encounters critical challenges from ethical restrictions and access to personalised patient data. The use of patient-derived cells and tissues for research has strict ethical restrictions, and the volume of eligible patients is insufficient for research requirements. The creation of personalised organ chips requires the simulation of multiple environmental parameters from the patient’s body which are patient-specific, such as immune levels and the degree of local vascularisation (which affects drug absorption and use). Finally, translating the findings of OC into the patient’s body is also a problem that needs to be considered. Determining what proportion of the obtained precise treatment plan should be scaled up to the human body and collecting data on the patients’ absorption and metabolism levels are obstacles to the future development of personalised OC. The establishment of a complete patient body-on-a-chip can be a solution.

### 4.2. Chronic-Wound-on-a-Chip Model

Current models for chronic wound research mainly 2D cellular scratches, 3D skin equivalents, and animal models. It is difficult to heal wounds formed by multiple complex factors acting on the wound surface, mainly complex and long-standing inflammatory reactions. Including multiple skin structures in two dimensions is difficult, and the usability of the study results is limited. Although 3D skin equivalents can present similar skin trauma structures to some extent, they still lack in vivo changes in environmental parameters such as changes in chemical gradients. Researchers have created a trauma microarray model containing blood vessels, immune cells, and dermal structures that allows cell-to-cell interactions to explore the use of anti-inflammatory drugs under inflammatory conditions [169]. We believe that such a chip model is associated with acute trauma, while chronic trauma has more complex cellular components, more difficult-to-control inflammatory conditions, and even specific requirements for blood supply, such as ischaemic chronic trauma and diabetic foot ulcers. Based on this model, by adding multiple cellular components and controlling the proportion of inflammatory cells and the culture fluid composition, we expect to establish a microarray model for chronic wounds.

For example, diabetic foot ulcer (DFU) chip models have not yet been fully established; however, the establishment of in vitro 3D DFU models can be a good inspiration for DFU chip models. Some researchers state that the ideal DFU chip model should embed diseased cells in the scaffold, promote the formation of diseased ECM, and actively elicit immune responses [170]. In the 3D DFU model, patient-derived cells were widely selected for tissue construction and their use slowed down the healing of the model wounds, including fibroblasts, keratin-forming cells, and monocytes. Studies have shown that there is also a large gap in the activity and polarisation of cells extracted in the middle of the wounds, at the edges of the wounds, and in healthy sites [171,172,173,174]. These cells are cultured in a high-glucose medium that mimics the high-sugar environment in vivo [171]. By integrating existing 3D models into a microfluidic platform that controls the cell source and culture fluid composition to provide the correct proportion of immune cells, DFU microarray models can be built.

In addition to the existing approach of integrating 3D chronic and acute wound models to form chronic-wound-on-a-chip models, the formation of a chip model of a chronic wound is based on a normal full-layer skin-on-a-chip, which is treated with a drug such as mercaptosuccinic acid (MSA) [175]. The same design can be used for other types of chronic wounds.

### 4.3. Skin Repair

The establishment of chronic-wound-on-a-chip models helps us study the complex inflammatory situation of chronic wounds, explore active and effective treatment methods, and promote faster healing. However, for difficult-to-heal wounds, the pathological mechanism is very clear, but it is very difficult to complete the healing process, such as in large deep burn wounds.

In the treatment of deep burn wounds, the ultimate goal is to repair the wound; however, patients with deep burns have skin defects and damage to the dermis that prevents spontaneous re-formation of skin to complete healing. Currently, deep burn wounds are often covered with autologous micro-dermis grafts or dECM or hydrogel scaffolds containing mesenchymal stem cells, in the hope of generating good skin coverage. This method is yet to be explored, and autologous skin sources are very limited for patients with large deep burns. We believe that OC can solve this problem by culturing suitable skin tissues in vitro as a therapeutic resource to be transplanted to the wound surface to complete repair. On the one hand, cells of patient origin can be selected for in vitro culture to get rid of the limitation of insufficient resources, and, on the other hand, excellent skin structures can be formed by in vitro 3D bioprinting and cultured for a long-time using microfluidics, which can increase the vascular component and improve the survival rate of the incoming skin tissue in the wound base. As mentioned above, the skin-on-a-chip model can be cultured on a large scale and transplanted in vivo for surface wound repair.

In addition, missing skin attachments can be repaired to achieve a high level of wound repair. Hair follicles are important accessories that protect the skin, regulate body temperature, and contain sweat glands, erector spinae, blood vessels, nerves, lymphatic vessels, and epithelial and dermal mesenchymal cells [176]. In the widely studied in vitro culture of hair follicles [177], the process of hair follicle formation in the embryo is simulated in vitro, where embryonic-derived epithelial and mesenchymal cells are co-cultured in a collagen scaffold and subsequently transplanted into the skin to form a certain structure and function of hair follicle tissue [178] (Figure 6(B1)). Researchers established a PDMS microwell array with a diameter of 1 mm and depth of 0.5 mm for in vitro large-scale culturing of hair follicle germ, in which mouse embryonic epithelial and mesenchymal cells were co-cultured in microwells, and the surface of the microwell was covered with colloidal material to maintain the cells in a fixed position. Hair follicle structure was formed after 18 days [179] (Figure 6(B2)). We consider this as a preliminary exploration of hair-follicle-on-a-chip, and the integration of microfluidic devices allows a better study of the effect of oxygen content on hair follicle germ development, as found in these studies. A full-layer skin chip model containing hair follicles, blood vessels, and nerves is possible in a large-scale culture chamber, allowing complete wound repair. Although it provides an ideal treatment for patients with large deep burns, there are still difficulties to be eliminated in the process of chip construction.

OCs are currently used in vitro; however, using them in vivo is equally necessary and advantageous. For example, most ischaemic ulcers develop into hard-to-heal wounds when they are treated because of the lack of blood infusion nutrients in the wounds. Inspired by the application of Ocs in vitro, the ulcerated area was closed and connected to a microfluidic and sensor system, allowing the formation of OC devices targeting wound tissue for culture, which can also cultivate cells with the assistance of tissue scaffolds in vivo, such as fibroblasts, epidermal cells, or even hair follicle germ missing from the wounds (Figure 6(B3)). The integrated sensor system can monitor the temperature, pH, cytokines, and other wound factors in real time, and the corresponding treatment can be applied using a micropump at any time. Such a concept has parallels with smart dressings, which have been developing rapidly in recent years. One of the best examples of smart dressings is the real-time monitoring and on-demand treatment of infected wounds, where the temperature of the wound may rise after infection and this information is transmitted through a temperature sensor, which controls the UV light-emitting diode on the dressing, so that the UV-sensitive antibiotic hydrogel dressing starts releasing antibiotics for antimicrobial treatment [180]. A smart dressing can only identify the presence of an infection, and not the degree of infection. It is of single use only and only decides whether to release antibiotics, while dose grasp is not possible with it. In contrast, in vivo OCs allow for a more comprehensive monitoring of wound conditions and more flexible and precise treatment delivery. This study also provides a novel treatment strategy for unhealed wounds.

The concept of in vivo use of organ chips is promising and can be applied not only for skin wound repair but also for tissue treatment in other areas. However, many factors still need to be considered during the implementation process (such as configuration of the culture medium, which promotes the growth of the target cell population without causing tissue hyperplasia), setting the pressure inside the chip system to avoid abnormal bleeding and ischaemia, and, most importantly, making a tight connection between the target site and the chip system, avoiding or eliminating the impact of tissue fluid exudation on the microfluidic system.

### 4.4. Challenges

Many challenges in production of OCs are as follows. First, OCs are difficult to produce and demanding to design. They are designed to mimic the most important aspects and typical features of the tissue, where the nature of problems encounter varies, as do the key aspects and typical features; however, their design is flexible and changeable. Therefore, designers must determine features operating on OCs in consideration during the design phase or when selecting a specific platform, such as channel diameter, angles and input/output ports, bubble traps, biomechanical forces, and the design of biosensors [15]. Most of the cells used in OCs are pluripotent cells and adult stem cells, and many of these cell-derived differentiated cells (e.g., cardiomyocytes) are phenotypically immature, have no standardised protocol for differentiation and maturation, and are difficult to replicate [43]. The choice of extracellular matrix in OCs is also challenging, and no standardised protocol is yet determined for its creation. Decellularized scaffolds or seeded cells in hydrogels can be used to build a suitable environment for cell growth, which is mostly influenced by the composition and arrangement of the scaffold. Therefore, it must be selected and designed carefully to promote appropriate tissue characteristics to form appropriately [181]. The nutrient preferences and aversions of tissues in OCs are different and require associated conservation of many different nutrients. A key challenge for the connected OCs tissue system is, therefore, the provision of this universal cell culture fluid or ‘blood mimic’. However, mixed media can solve the problem of a limited number of connected OCs, which is not applicable for connected multiple OCs, and the creation of a single-channel medium that can be added or updated over time is a potential solution that is as-of-yet unexplored [105,141]. OCs cannot replicate certain aspects of the body, and even the most complex MOC may miss some tissues, resulting in a series of OCs missing changes in human metabolism, such as diurnal changes [182], temperature changes [59,183], and changes in drug absorption due to endocrine changes. One solution is to create complex ‘micro formulations’ to deliver media at particular intervals to compensate for the missing organ [16]; however, this remains a challenge. Finally, the fabrication materials used to fabricate the OCs must be taken into account. Regardless of the material chosen for manufacturing, aspects such as its adsorption properties and biocompatibility should be carefully observed [56,64].

## 5. Summary

OCs have made drug development faster, economical, and more efficient; they have also enabled a deeper, more detailed understanding of diseases. They have provided novel and interesting approaches for disease treatment and prevention, especially with MOC systems formed by the integration of many OCs, such that their development from one to many can potentially realise body-on-a-chip (BOC) platforms. Although many applications of OCs have been revolutionary, it is believed that there are still more astounding applications to be discovered.

## Data Availability

Not applicable.

## References

[1] Arrowsmith J., Miller P. (2013). Trial watch: Phase II and phase III attrition rates 2011-2012. Nat. Rev. Drug Discov..

[2] Bhatia S.N., Ingber D.E. (2014). Microfluidic organs-on-chips. Nat. Biotechnol..

[3] Brajsa K., Vujasinovic I., Jelic D., Trzun M., Zlatar I., Karminski-Zamola G., Hranjec M. (2016). Antitumor activity of amidino-substituted benzimidazole and benzimidazo [1,2-a]quinoline derivatives tested in 2D and 3D cell culture systems. J. Enzyme Inhib. Med. Chem..

[4] Van de Stolpe A., den Toonder J. (2013). Workshop meeting report Organs-on-Chips: Human disease models. Lab Chip.

[5] Rossi G., Manfrin A., Lutolf M.P. (2018). Progress and potential in organoid research. Nat. Rev. Genet..

[6] Clevers H. (2016). Modeling Development and Disease with Organoids. Cell.

[7] Duval K., Grover H., Han L.H., Mou Y., Pegoraro A.F., Fredberg J., Chen Z. (2017). Modeling Physiological Events in 2D vs. 3D Cell Culture. Physiology.

[8] Ryan S.L., Baird A.M., Vaz G., Urquhart A.J., Senge M., Richard D.J., O’Byrne K.J., Davies A.M. (2016). Drug Discovery Approaches Utilizing Three-Dimensional Cell Culture. Assay Drug Dev. Technol..

[9] Jia X., Hua C., Yang F., Li X., Zhao P., Zhou F., Lu Y., Xing M., Lu G. (2023). Hydrophobic aerogel-modified hemostatic gauze with thermal management performance. Bioact. Mater..

[10] Ahadian S., Civitarese R., Bannerman D., Mohammadi M.H., Lu R., Wang E., Davenport-Huyer L., Lai B., Zhang B., Zhao Y. (2018). Organ-On-A-Chip Platforms: A Convergence of Advanced Materials, Cells, and Microscale Technologies. Adv. Health Mater..

[11] Huh D., Hamilton G.A., Ingber D.E. (2011). From 3D cell culture to organs-on-chips. Trends Cell Biol..

[12] Harink B., Le Gac S., Truckenmuller R., van Blitterswijk C., Habibovic P. (2013). Regeneration-on-a-chip? The perspectives on use of microfluidics in regenerative medicine. Lab Chip.

[13] Jaalouk D.E., Lammerding J. (2009). Mechanotransduction gone awry. Nat. Rev. Mol. Cell Biol..

[14] Thompson C.L., Fu S., Knight M.M., Thorpe S.D. (2020). Mechanical Stimulation: A Crucial Element of Organ-on-Chip Models. Front. Bioeng. Biotechnol..

[15] Huh D., Matthews B.D., Mammoto A., Montoya-Zavala M., Hsin H.Y., Ingber D.E. (2010). Reconstituting organ-level lung functions on a chip. Science.

[16] Low L.A., Mummery C., Berridge B.R., Austin C.P., Tagle D.A. (2021). Organs-on-chips: Into the next decade. Nat. Rev. Drug Discov..

[17] Zhu Y., Yin F., Wang H., Wang L., Yuan J., Qin J. (2018). Placental Barrier-on-a-Chip: Modeling Placental Inflammatory Responses to Bacterial Infection. ACS Biomater. Sci. Eng..

[18] Ahn S.I., Sei Y.J., Park H.J., Kim J., Ryu Y., Choi J.J., Sung H.J., MacDonald T.J., Levey A.I., Kim Y. (2020). Microengineered human blood-brain barrier platform for understanding nanoparticle transport mechanisms. Nat. Commun..

[19] Maschmeyer I., Lorenz A.K., Schimek K., Hasenberg T., Ramme A.P., Hubner J., Lindner M., Drewell C., Bauer S., Thomas A. (2015). A four-organ-chip for interconnected long-term co-culture of human intestine, liver, skin and kidney equivalents. Lab Chip.

[20] Wieringa P.A., Goncalves de Pinho A.R., Micera S., van Wezel R.J.A., Moroni L. (2018). Biomimetic Architectures for Peripheral Nerve Repair: A Review of Biofabrication Strategies. Adv. Health Mater..

[21] Liaw C.Y., Ji S., Guvendiren M. (2018). Engineering 3D Hydrogels for Personalized In Vitro Human Tissue Models. Adv. Health Mater..

[22] Luan Z., Liu S., Wang W., Xu K., Ye S., Dan R., Zhang H., Shu Z., Wang T., Fan C. (2022). Aligned nanofibrous collagen membranes from fish swim bladder as a tough and acid-resistant suture for pH-regulated stomach perforation and tendon rupture. Biomater. Res..

[23] Yang J., Zhang Y.S., Yue K., Khademhosseini A. (2017). Cell-laden hydrogels for osteochondral and cartilage tissue engineering. Acta Biomater..

[24] Arık Y.B., de Sa Vivas A., Laarveld D., van Laar N., Gemser J., Visscher T., van den Berg A., Passier R., van der Meer A.D. (2021). Collagen I Based Enzymatically Degradable Membranes for Organ-on-a-Chip Barrier Models. ACS Biomater. Sci. Eng..

[25] Zamprogno P., Thoma G., Cencen V., Ferrari D., Putz B., Michler J., Fantner G.E., Guenat O.T. (2021). Mechanical Properties of Soft Biological Membranes for Organ-on-a-Chip Assessed by Bulge Test and AFM. ACS Biomater. Sci. Eng..

[26] Li W., Zhang L., Ge X., Xu B., Zhang W., Qu L., Choi C.H., Xu J., Zhang A., Lee H. (2018). Microfluidic fabrication of microparticles for biomedical applications. Chem. Soc. Rev..

[27] He T., Wang W., Chen B., Wang J., Liang Q., Chen B. (2020). 5-Fluorouracil monodispersed chitosan microspheres: Microfluidic chip fabrication with crosslinking, characterization, drug release and anticancer activity. Carbohydr. Polym..

[28] Agarwal A., Farouz Y., Nesmith A.P., Deravi L.F., McCain M.L., Parker K.K. (2013). Micropatterning Alginate Substrates for in vitro Cardiovascular Muscle on a Chip. Adv. Funct. Mater..

[29] Jiang W., Li M., Chen Z., Leong K.W. (2016). Cell-laden microfluidic microgels for tissue regeneration. Lab Chip.

[30] Lee K.Y., Mooney D.J. (2012). Alginate: Properties and biomedical applications. Prog. Polym. Sci..

[31] Yue K., Trujillo-de Santiago G., Alvarez M.M., Tamayol A., Annabi N., Khademhosseini A. (2015). Synthesis, properties, and biomedical applications of gelatin methacryloyl (GelMA) hydrogels. Biomaterials.

[32] Li X., Luo Y., Yang F., Chu G., Li L., Diao L., Jia X., Yu C., Wu X., Zhong W. (2023). In situ-formed micro silk fibroin composite sutures for pain management and anti-infection. Compos. Part B Eng..

[33] Darabi M.A., Khosrozadeh A., Wang Y., Ashammakhi N., Alem H., Erdem A., Chang Q., Xu K., Liu Y., Luo G. (2020). An Alkaline Based Method for Generating Crystalline, Strong, and Shape Memory Polyvinyl Alcohol Biomaterials. Adv. Sci..

[34] Abdallah M., Martin M., El Tahchi M.R., Balme S., Faour W.H., Varga B., Cloitre T., Páll O., Cuisinier F.J.G., Gergely C. (2019). Influence of Hydrolyzed Polyacrylamide Hydrogel Stiffness on Podocyte Morphology, Phenotype, and Mechanical Properties. ACS Appl. Mater. Interfaces.

[35] Wang Z., Abdulla R., Parker B., Samanipour R., Ghosh S., Kim K. (2015). A simple and high-resolution stereolithography-based 3D bioprinting system using visible light crosslinkable bioinks. Biofabrication.

[36] Dikovsky D., Bianco-Peled H., Seliktar D. (2006). The effect of structural alterations of PEG-fibrinogen hydrogel scaffolds on 3-D cellular morphology and cellular migration. Biomaterials.

[37] Humayun M., Chow C.W., Young E.W.K. (2018). Microfluidic lung airway-on-a-chip with arrayable suspended gels for studying epithelial and smooth muscle cell interactions. Lab Chip.

[38] Shim K.Y., Lee D., Han J., Nguyen N.T., Park S., Sung J.H. (2017). Microfluidic gut-on-a-chip with three-dimensional villi structure. Biomed. Microdevices.

[39] Zhang X., Li L., Luo C. (2016). Gel integration for microfluidic applications. Lab Chip.

[40] Wang Y.I., Abaci H.E., Shuler M.L. (2017). Microfluidic blood-brain barrier model provides in vivo-like barrier properties for drug permeability screening. Biotechnol. Bioeng..

[41] Hare D., Collins S., Cuddington B., Mossman K. (2016). The Importance of Physiologically Relevant Cell Lines for Studying Virus-Host Interactions. Viruses.

[42] Pan C., Kumar C., Bohl S., Klingmueller U., Mann M. (2009). Comparative proteomic phenotyping of cell lines and primary cells to assess preservation of cell type-specific functions. Mol. Cell Proteom..

[43] Wnorowski A., Yang H., Wu J.C. (2019). Progress, obstacles, and limitations in the use of stem cells in organ-on-a-chip models. Adv. Drug Deliv. Rev..

[44] Mummery C. (2004). Stem cell research: Immortality or a healthy old age?. Eur. J. Endocrinol..

[45] Takahashi K., Yamanaka S. (2006). Induction of pluripotent stem cells from mouse embryonic and adult fibroblast cultures by defined factors. Cell.

[46] Li X., Brooks J.C., Hu J., Ford K.I., Easley C.J. (2017). 3D-templated, fully automated microfluidic input/output multiplexer for endocrine tissue culture and secretion sampling. Lab Chip.

[47] Maschmeyer I., Hasenberg T., Jaenicke A., Lindner M., Lorenz A.K., Zech J., Garbe L.A., Sonntag F., Hayden P., Ayehunie S. (2015). Chip-based human liver-intestine and liver-skin co-cultures--A first step toward systemic repeated dose substance testing in vitro. Eur. J. Pharm. Biopharm..

[48] Wang Y., Ahmad A.A., Sims C.E., Magness S.T., Allbritton N.L. (2014). In vitro generation of colonic epithelium from primary cells guided by microstructures. Lab Chip.

[49] Benam K.H., Villenave R., Lucchesi C., Varone A., Hubeau C., Lee H.H., Alves S.E., Salmon M., Ferrante T.C., Weaver J.C. (2016). Small airway-on-a-chip enables analysis of human lung inflammation and drug responses in vitro. Nat. Methods.

[50] Nawroth J., Rogal J., Weiss M., Brucker S.Y., Loskill P. (2018). Organ-on-a-Chip Systems for Women’s Health Applications. Adv. Health Mater..

[51] Simoncini T., Giannini A., Genazzani A.R. (2017). The Long-Term Cardiovascular Risks Associated with Amenorrhea. Frontiers in Gynecological Endocrinology.

[52] Werling D.M., Geschwind D.H. (2013). Sex differences in autism spectrum disorders. Curr. Opin. Neurol..

[53] Altemus M., Sarvaiya N., Neill Epperson C. (2014). Sex differences in anxiety and depression clinical perspectives. Front. Neuroendocrinol..

[54] Wenger N.K., Ouyang P., Miller V.M., Bairey Merz C.N. (2016). Strategies and Methods for Clinical Scientists to Study Sex-Specific Cardiovascular Health and Disease in Women. J. Am. Coll. Cardiol..

[55] Klein S.L., Flanagan K.L. (2016). Sex differences in immune responses. Nat. Rev. Immunol..

[56] Ding C., Chen X., Kang Q., Yan X. (2020). Biomedical Application of Functional Materials in Organ-on-a-Chip. Front. Bioeng Biotechnol..

[57] McDonald J.C., Whitesides G.M. (2002). Poly(dimethylsiloxane) as a material for fabricating microfluidic devices. Acc. Chem. Res..

[58] Liu Y., Guan G., Li Y., Tan J., Cheng P., Yang M., Li B., Wang Q., Zhong W., Mequanint K. (2022). Gelation of highly entangled hydrophobic macromolecular fluid for ultrastrong underwater in situ fast tissue adhesion. Sci. Adv..

[59] Bhattacharjee N., Urrios A., Kang S., Folch A. (2016). The upcoming 3D-printing revolution in microfluidics. Lab Chip.

[60] Su X., Young E.W., Underkofler H.A., Kamp T.J., January C.T., Beebe D.J. (2011). Microfluidic cell culture and its application in high-throughput drug screening: Cardiotoxicity assay for hERG channels. J. Biomol. Screen.

[61] Regehr K.J., Domenech M., Koepsel J.T., Carver K.C., Ellison-Zelski S.J., Murphy W.L., Schuler L.A., Alarid E.T., Beebe D.J. (2009). Biological implications of polydimethylsiloxane-based microfluidic cell culture. Lab Chip.

[62] Paoli R., Di Giuseppe D., Badiola-Mateos M., Martinelli E., Lopez-Martinez M.J., Samitier J. (2021). Rapid Manufacturing of Multilayered Microfluidic Devices for Organ on a Chip Applications. Sensors.

[63] Zhang B., Lai B.F.L., Xie R., Davenport Huyer L., Montgomery M., Radisic M. (2018). Microfabrication of AngioChip, a biodegradable polymer scaffold with microfluidic vasculature. Nat. Protoc..

[64] Ren K., Zhou J., Wu H. (2013). Materials for microfluidic chip fabrication. Acc. Chem. Res..

[65] Piruska A., Nikcevic I., Lee S.H., Ahn C., Heineman W.R., Limbach P.A., Seliskar C.J. (2005). The autofluorescence of plastic materials and chips measured under laser irradiation. Lab Chip.

[66] Miller P.G., Shuler M.L. (2016). Design and demonstration of a pumpless 14 compartment microphysiological system. Biotechnol. Bioeng..

[67] Hirama H., Satoh T., Sugiura S., Shin K., Onuki-Nagasaki R., Kanamori T., Inoue T. (2019). Glass-based organ-on-a-chip device for restricting small molecular absorption. J. Biosci. Bioeng..

[68] Xu K., Wu X., Zhang X., Xing M. (2022). Bridging wounds: Tissue adhesives’ essential mechanisms, synthesis and characterization, bioinspired adhesives and future perspectives. Burn. Trauma.

[69] Huang Y., Fan C., Liu Y., Yang L., Hu W., Liu S., Wang T., Shu Z., Li B., Xing M. (2022). Nature-Derived Okra Gel as Strong Hemostatic Bioadhesive in Human Blood, Liver, and Heart Trauma of Rabbits and Dogs. Adv. Health Mater..

[70] Huang G., Li F., Zhao X., Ma Y., Li Y., Lin M., Jin G., Lu T.J., Genin G.M., Xu F. (2017). Functional and Biomimetic Materials for Engineering of the Three-Dimensional Cell Microenvironment. Chem. Rev..

[71] Huang Y., Cai D., Chen P. (2011). Micro- and nanotechnologies for study of cell secretion. Anal. Chem..

[72] Huh D., Leslie D.C., Matthews B.D., Fraser J.P., Jurek S., Hamilton G.A., Thorneloe K.S., McAlexander M.A., Ingber D.E. (2012). A human disease model of drug toxicity-induced pulmonary edema in a lung-on-a-chip microdevice. Sci. Transl. Med..

[73] Jang K.J., Suh K.Y. (2010). A multi-layer microfluidic device for efficient culture and analysis of renal tubular cells. Lab Chip.

[74] Chapanian R., Amsden B.G. (2010). Combined and sequential delivery of bioactive VEGF165 and HGF from poly(trimethylene carbonate) based photo-cross-linked elastomers. J. Control Release.

[75] Darabi M.A., Khosrozadeh A., Mbeleck R., Liu Y., Chang Q., Jiang J., Cai J., Wang Q., Luo G., Xing M. (2017). Skin-Inspired Multifunctional Autonomic-Intrinsic Conductive Self-Healing Hydrogels with Pressure Sensitivity, Stretchability, and 3D Printability. Adv. Mater..

[76] Chang Q., He Y., Liu Y., Zhong W., Wang Q., Lu F., Xing M. (2020). Protein Gel Phase Transition: Toward Superiorly Transparent and Hysteresis-Free Wearable Electronics. Adv. Funct. Mater..

[77] Matai I., Kaur G., Seyedsalehi A., McClinton A., Laurencin C.T. (2020). Progress in 3D bioprinting technology for tissue/organ regenerative engineering. Biomaterials.

[78] Mandrycky C., Wang Z., Kim K., Kim D.H. (2016). 3D bioprinting for engineering complex tissues. Biotechnol. Adv..

[79] Sill T.J., von Recum H.A. (2008). Electrospinning: Applications in drug delivery and tissue engineering. Biomaterials.

[80] Polte T.R., Eichler G.S., Wang N., Ingber D.E. (2004). Extracellular matrix controls myosin light chain phosphorylation and cell contractility through modulation of cell shape and cytoskeletal prestress. Am. J. Physiol. Cell Physiol..

[81] Takayama S., Ostuni E., LeDuc P., Naruse K., Ingber D.E., Whitesides G.M. (2001). Subcellular positioning of small molecules. Nature.

[82] Andersson H., van den Berg A. (2004). Microfabrication and microfluidics for tissue engineering: State of the art and future opportunities. Lab Chip.

[83] Ho C.T., Lin R.Z., Chang W.Y., Chang H.Y., Liu C.H. (2006). Rapid heterogeneous liver-cell on-chip patterning via the enhanced field-induced dielectrophoresis trap. Lab Chip.

[84] Daley W.P., Peters S.B., Larsen M. (2008). Extracellular matrix dynamics in development and regenerative medicine. J. Cell Sci..

[85] Morrison S.J., Scadden D.T. (2014). The bone marrow niche for haematopoietic stem cells. Nature.

[86] Lee K., Silva E.A., Mooney D.J. (2011). Growth factor delivery-based tissue engineering: General approaches and a review of recent developments. J. R. Soc. Interface.

[87] Sharifi F., Htwe S.S., Righi M., Liu H., Pietralunga A., Yesil-Celiktas O., Maharjan S., Cha B.H., Shin S.R., Dokmeci M.R. (2019). A Foreign Body Response-on-a-Chip Platform. Adv. Health Mater..

[88] Beckwitt C.H., Clark A.M., Wheeler S., Taylor D.L., Stolz D.B., Griffith L., Wells A. (2018). Liver ‘organ on a chip’. Exp. Cell Res..

[89] Li X., George S.M., Vernetti L., Gough A.H., Taylor D.L. (2018). A glass-based, continuously zonated and vascularized human liver acinus microphysiological system (vLAMPS) designed for experimental modeling of diseases and ADME/TOX. Lab Chip.

[90] Subramanian A., Krishnan U.M., Sethuraman S. (2009). Development of biomaterial scaffold for nerve tissue engineering: Biomaterial mediated neural regeneration. J. Biomed. Sci..

[91] Nunes S.S., Miklas J.W., Liu J., Aschar-Sobbi R., Xiao Y., Zhang B., Jiang J., Masse S., Gagliardi M., Hsieh A. (2013). Biowire: A platform for maturation of human pluripotent stem cell-derived cardiomyocytes. Nat. Methods.

[92] Galie P.A., Nguyen D.H., Choi C.K., Cohen D.M., Janmey P.A., Chen C.S. (2014). Fluid shear stress threshold regulates angiogenic sprouting. Proc. Natl. Acad. Sci. USA.

[93] Weng Y.S., Chang S.F., Shih M.C., Tseng S.H., Lai C.H. (2017). Scaffold-Free Liver-On-A-Chip with Multiscale Organotypic Cultures. Adv. Mater..

[94] Rodrigues R.O., Sousa P.C., Gaspar J., Banobre-Lopez M., Lima R., Minas G. (2020). Organ-on-a-Chip: A Preclinical Microfluidic Platform for the Progress of Nanomedicine. Small.

[95] Zhang Y.S., Aleman J., Shin S.R., Kilic T., Kim D., Mousavi Shaegh S.A., Massa S., Riahi R., Chae S., Hu N. (2017). Multisensor-integrated organs-on-chips platform for automated and continual in situ monitoring of organoid behaviors. Proc. Natl. Acad. Sci. USA.

[96] Kieninger J., Weltin A., Flamm H., Urban G.A. (2018). Microsensor systems for cell metabolism—From 2D culture to organ-on-chip. Lab Chip.

[97] Cohen Z.J., Haxha S., Aggoun A. (2016). Pulse oximetry optical sensor using oxygen-bound haemoglobin. Opt. Express.

[98] Rumpler M., Hajnsek M., Baumann P., Pieber T.R., Klimant I. (2018). Monitoring tissue oxygen heterogeneities and their influence on optical glucose measurements in an animal model. J. Clin. Monit. Comput..

[99] Shin S.R., Kilic T., Zhang Y.S., Avci H., Hu N., Kim D., Branco C., Aleman J., Massa S., Silvestri A. (2017). Label-Free and Regenerative Electrochemical Microfluidic Biosensors for Continual Monitoring of Cell Secretomes. Adv. Sci..

[100] Riahi R., Shaegh S.A., Ghaderi M., Zhang Y.S., Shin S.R., Aleman J., Massa S., Kim D., Dokmeci M.R., Khademhosseini A. (2016). Automated microfluidic platform of bead-based electrochemical immunosensor integrated with bioreactor for continual monitoring of cell secreted biomarkers. Sci. Rep..

[101] Xu C., Jiang D., Ge Y., Huang L., Xiao Y., Ren X., Liu X., Zhang Q., Wang Y. (2022). A PEDOT:PSS conductive hydrogel incorporated with Prussian blue nanoparticles for wearable and noninvasive monitoring of glucose. Chem. Eng. J..

[102] Odijk M., van der Meer A.D., Levner D., Kim H.J., van der Helm M.W., Segerink L.I., Frimat J.P., Hamilton G.A., Ingber D.E., van den Berg A. (2015). Measuring direct current trans-epithelial electrical resistance in organ-on-a-chip microsystems. Lab Chip.

[103] Chen W.L.K., Edington C., Suter E., Yu J., Velazquez J.J., Velazquez J.G., Shockley M., Large E.M., Venkataramanan R., Hughes D.J. (2017). Integrated gut/liver microphysiological systems elucidates inflammatory inter-tissue crosstalk. Biotechnol. Bioeng..

[104] Cao U.M.N., Zhang Y., Chen J., Sayson D., Pillai S., Tran S.D. (2023). Microfluidic Organ-on-A-chip: A Guide to Biomaterial Choice and Fabrication. Int. J. Mol. Sci..

[105] Chang S.Y., Weber E.J., Sidorenko V.S., Chapron A., Yeung C.K., Gao C., Mao Q., Shen D., Wang J., Rosenquist T.A. (2017). Human liver-kidney model elucidates the mechanisms of aristolochic acid nephrotoxicity. JCI Insight.

[106] Yang F., Cohen R.N., Brey E.M. (2020). Optimization of Co-Culture Conditions for a Human Vascularized Adipose Tissue Model. Bioengineering.

[107] Zhang C., Zhao Z., Abdul Rahim N.A., van Noort D., Yu H. (2009). Towards a human-on-chip: Culturing multiple cell types on a chip with compartmentalized microenvironments. Lab Chip.

[108] Jalili-Firoozinezhad S., Gazzaniga F.S., Calamari E.L., Camacho D.M., Fadel C.W., Bein A., Swenor B., Nestor B., Cronce M.J., Tovaglieri A. (2019). A complex human gut microbiome cultured in an anaerobic intestine-on-a-chip. Nat. Biomed. Eng..

[109] Abbott N.J., Patabendige A.A., Dolman D.E., Yusof S.R., Begley D.J. (2010). Structure and function of the blood-brain barrier. Neurobiol. Dis..

[110] Park D., Lee J., Chung J.J., Jung Y., Kim S.H. (2020). Integrating Organs-on-Chips: Multiplexing, Scaling, Vascularization, and Innervation. Trends Biotechnol..

[111] Staicu C.E., Jipa F., Axente E., Radu M., Radu B.M., Sima F. (2021). Lab-on-a-Chip Platforms as Tools for Drug Screening in Neuropathologies Associated with Blood-Brain Barrier Alterations. Biomolecules.

[112] Fung K.Y., Wang C., Nyegaard S., Heit B., Fairn G.D., Lee W.L. (2017). SR-BI Mediated Transcytosis of HDL in Brain Microvascular Endothelial Cells Is Independent of Caveolin, Clathrin, and PDZK1. Front. Physiol..

[113] Wang H., Eckel R.H. (2014). What are lipoproteins doing in the brain?. Trends Endocrinol. Metab..

[114] Blundell C., Yi Y.S., Ma L., Tess E.R., Farrell M.J., Georgescu A., Aleksunes L.M., Huh D. (2018). Placental Drug Transport-on-a-Chip: A Microengineered In Vitro Model of Transporter-Mediated Drug Efflux in the Human Placental Barrier. Adv. Health Mater..

[115] Young R.E., Huh D.D. (2021). Organ-on-a-chip technology for the study of the female reproductive system. Adv. Drug Deliv. Rev..

[116] Richardson L., Kim S., Menon R., Han A. (2020). Organ-On-Chip Technology: The Future of Feto-Maternal Interface Research?. Front. Physiol..

[117] Richardson L., Vargas G., Brown T., Ochoa L., Trivedi J., Kacerovsky M., Lappas M., Menon R. (2017). Redefining 3Dimensional placental membrane microarchitecture using multiphoton microscopy and optical clearing. Placenta.

[118] Gnecco J.S., Anders A.P., Cliffel D., Pensabene V., Rogers L.M., Osteen K., Aronoff D.M. (2017). Instrumenting a Fetal Membrane on a Chip as Emerging Technology for Preterm Birth Research. Curr. Pharm. Des..

[119] Arver S., Stief C., de la Rosette J., Jones T.H., Neijber A., Carrara D. (2018). A new 2% testosterone gel formulation: A comparison with currently available topical preparations. Andrology.

[120] Rasmussen S., Horkan K.H., Kotler M. (2018). Pharmacokinetic Evaluation of Two Nicotine Patches in Smokers. Clin. Pharmacol. Drug Dev..

[121] Tarnoki-Zach J., Mehes E., Varga-Medveczky Z., Isai D.G., Barany N., Bugyik E., Revesz Z., Paku S., Erdo F., Czirok A. (2021). Development and Evaluation of a Human Skin Equivalent in a Semiautomatic Microfluidic Diffusion Chamber. Pharmaceutics.

[122] Sriram G., Alberti M., Dancik Y., Wu B., Wu R., Feng Z., Ramasamy S., Bigliardi P.L., Bigliardi-Qi M., Wang Z. (2018). Full-thickness human skin-on-chip with enhanced epidermal morphogenesis and barrier function. Mater. Today.

[123] Lukacs B., Bajza A., Kocsis D., Csorba A., Antal I., Ivan K., Laki A.J., Erdo F. (2019). Skin-on-a-Chip Device for Ex Vivo Monitoring of Transdermal Delivery of Drugs-Design, Fabrication, and Testing. Pharmaceutics.

[124] Bajza A., Kocsis D., Berezvai O., Laki A.J., Lukacs B., Imre T., Ivan K., Szabo P., Erdo F. (2020). Verification of P-Glycoprotein Function at the Dermal Barrier in Diffusion Cells and Dynamic “Skin-On-A-Chip” Microfluidic Device. Pharmaceutics.

[125] Jeong S., Kim J., Jeon H.M., Kim K., Sung G.Y. (2021). Development of an Aged Full-Thickness Skin Model Using Flexible Skin-on-a-Chip Subjected to Mechanical Stimulus Reflecting the Circadian Rhythm. Int. J. Mol. Sci..

[126] Jeon B., Lee G., Wufuer M., Huang Y., Choi Y., Kim S., Choi T.H. (2020). Enhanced predictive capacity using dual-parameter chip model that simulates physiological skin irritation. Toxicol. In Vitro.

[127] Mori N., Morimoto Y., Takeuchi S. (2017). Skin integrated with perfusable vascular channels on a chip. Biomaterials.

[128] Zhang Q., Sito L., Mao M., He J., Zhang Y.S., Zhao X. (2018). Current advances in skin-on-a-chip models for drug testing. Microphysiol. Syst..

[129] Jungermann K., Kietzmann T. (2000). Oxygen: Modulator of metabolic zonation and disease of the liver. Hepatology.

[130] Lee-Montiel F.T., George S.M., Gough A.H., Sharma A.D., Wu J., DeBiasio R., Vernetti L.A., Taylor D.L. (2017). Control of oxygen tension recapitulates zone-specific functions in human liver microphysiology systems. Exp. Biol. Med..

[131] Sung J.H., Shuler M.L. (2009). A micro cell culture analog (microCCA) with 3-D hydrogel culture of multiple cell lines to assess metabolism-dependent cytotoxicity of anti-cancer drugs. Lab Chip.

[132] Cecen B., Karavasili C., Nazir M., Bhusal A., Dogan E., Shahriyari F., Tamburaci S., Buyukoz M., Kozaci L.D., Miri A.K. (2021). Multi-Organs-on-Chips for Testing Small-Molecule Drugs: Challenges and Perspectives. Pharmaceutics.

[133] Esch E.W., Bahinski A., Huh D. (2015). Organs-on-chips at the frontiers of drug discovery. Nat. Rev. Drug Discov..

[134] Bhise N.S., Ribas J., Manoharan V., Zhang Y.S., Polini A., Massa S., Dokmeci M.R., Khademhosseini A. (2014). Organ-on-a-chip platforms for studying drug delivery systems. J. Control Release.

[135] Herland A., Maoz B.M., Das D., Somayaji M.R., Prantil-Baun R., Novak R., Cronce M., Huffstater T., Jeanty S.S.F., Ingram M. (2020). Quantitative prediction of human pharmacokinetic responses to drugs via fluidically coupled vascularized organ chips. Nat. Biomed. Eng..

[136] Skardal A., Aleman J., Forsythe S., Rajan S., Murphy S., Devarasetty M., Pourhabibi Zarandi N., Nzou G., Wicks R., Sadri-Ardekani H. (2020). Drug compound screening in single and integrated multi-organoid body-on-a-chip systems. Biofabrication.

[137] Novak R., Ingram M., Marquez S., Das D., Delahanty A., Herland A., Maoz B.M., Jeanty S.S.F., Somayaji M.R., Burt M. (2020). Robotic fluidic coupling and interrogation of multiple vascularized organ chips. Nat. Biomed. Eng..

[138] Leclerc E., Hamon J., Bois F.Y. (2016). Investigation of ifosfamide and chloroacetaldehyde renal toxicity through integration of in vitro liver-kidney microfluidic data and pharmacokinetic-system biology models. J. Appl. Toxicol..

[139] Wagner I., Materne E.M., Brincker S., Sussbier U., Fradrich C., Busek M., Sonntag F., Sakharov D.A., Trushkin E.V., Tonevitsky A.G. (2013). A dynamic multi-organ-chip for long-term cultivation and substance testing proven by 3D human liver and skin tissue co-culture. Lab Chip.

[140] Si L., Bai H., Rodas M., Cao W., Oh C.Y., Jiang A., Moller R., Hoagland D., Oishi K., Horiuchi S. (2021). A human-airway-on-a-chip for the rapid identification of candidate antiviral therapeutics and prophylactics. Nat. Biomed. Eng..

[141] Phan D.T.T., Wang X., Craver B.M., Sobrino A., Zhao D., Chen J.C., Lee L.Y.N., George S.C., Lee A.P., Hughes C.C.W. (2017). A vascularized and perfused organ-on-a-chip platform for large-scale drug screening applications. Lab Chip.

[142] Lee J., Mehrotra S., Zare-Eelanjegh E., Rodrigues R.O., Akbarinejad A., Ge D., Amato L., Kiaee K., Fang Y., Rosenkranz A. (2021). A Heart-Breast Cancer-on-a-Chip Platform for Disease Modeling and Monitoring of Cardiotoxicity Induced by Cancer Chemotherapy. Small.

[143] Wang X., Phan D.T., Sobrino A., George S.C., Hughes C.C., Lee A.P. (2016). Engineering anastomosis between living capillary networks and endothelial cell-lined microfluidic channels. Lab Chip.

[144] Sobrino A., Phan D.T., Datta R., Wang X., Hachey S.J., Romero-Lopez M., Gratton E., Lee A.P., George S.C., Hughes C.C. (2016). 3D microtumors in vitro supported by perfused vascular networks. Sci. Rep..

[145] Hwang S.H., Lee S., Park J.Y., Jeon J.S., Cho Y.J., Kim S. (2021). Potential of Drug Efficacy Evaluation in Lung and Kidney Cancer Models Using Organ-on-a-Chip Technology. Micromachines.

[146] Berzina S., Harrison A., Taly V., Xiao W. (2021). Technological Advances in Tumor-On-Chip Technology: From Bench to Bedside. Cancers.

[147] Caballero D., Kaushik S., Correlo V.M., Oliveira J.M., Reis R.L., Kundu S.C. (2017). Organ-on-chip models of cancer metastasis for future personalized medicine: From chip to the patient. Biomaterials.

[148] Kilickap S., Barista I., Akgul E., Aytemir K., Aksoyek S., Aksoy S., Celik I., Kes S., Tekuzman G. (2005). cTnT can be a useful marker for early detection of anthracycline cardiotoxicity. Ann. Oncol..

[149] Simoes R., Silva L.M., Cruz A., Fraga V.G., de Paula Sabino A., Gomes K.B. (2018). Troponin as a cardiotoxicity marker in breast cancer patients receiving anthracycline-based chemotherapy: A narrative review. Biomed. Pharmacother..

[150] Sieber S., Wirth L., Cavak N., Koenigsmark M., Marx U., Lauster R., Rosowski M. (2018). Bone marrow-on-a-chip: Long-term culture of human haematopoietic stem cells in a three-dimensional microfluidic environment. J. Tissue Eng. Regen. Med..

[151] Arai F., Suda T. (2007). Maintenance of quiescent hematopoietic stem cells in the osteoblastic niche. Ann. N. Y. Acad. Sci..

[152] Abdallah B.M., Kassem M. (2008). Human mesenchymal stem cells: From basic biology to clinical applications. Gene Ther..

[153] Lilly A.J., Johnson W.E., Bunce C.M. (2011). The haematopoietic stem cell niche: New insights into the mechanisms regulating haematopoietic stem cell behaviour. Stem Cells Int..

[154] Didwania M., Didwania A., Mehta G., Basak G.W., Yasukawa S., Takayama S., de Necochea-Campion R., Srivastava A., Carrier E. (2011). Artificial hematopoietic stem cell niche: Bioscaffolds to microfluidics to mathematical simulations. Curr. Top Med. Chem..

[155] Zhang B., Montgomery M., Chamberlain M.D., Ogawa S., Korolj A., Pahnke A., Wells L.A., Masse S., Kim J., Reis L. (2016). Biodegradable scaffold with built-in vasculature for organ-on-a-chip engineering and direct surgical anastomosis. Nat. Mater..

[156] Langer R., Vacanti J.P. (1993). Tissue engineering. Science.

[157] Rafii S., Lyden D. (2003). Therapeutic stem and progenitor cell transplantation for organ vascularization and regeneration. Nat. Med..

[158] Breitbach M., Bostani T., Roell W., Xia Y., Dewald O., Nygren J.M., Fries J.W., Tiemann K., Bohlen H., Hescheler J. (2007). Potential risks of bone marrow cell transplantation into infarcted hearts. Blood.

[159] Badylak S.F., Gilbert T.W. (2008). Immune response to biologic scaffold materials. Semin. Immunol..

[160] Skardal A., Murphy S.V., Devarasetty M., Mead I., Kang H.W., Seol Y.J., Shrike Zhang Y., Shin S.R., Zhao L., Aleman J. (2017). Multi-tissue interactions in an integrated three-tissue organ-on-a-chip platform. Sci. Rep..

[161] Guenat O.T., Geiser T., Berthiaume F. (2020). Clinically Relevant Tissue Scale Responses as New Readouts from Organs-on-a-Chip for Precision Medicine. Annu. Rev. Anal. Chem..

[162] van den Berg A., Mummery C.L., Passier R., van der Meer A.D. (2019). Personalised organs-on-chips: Functional testing for precision medicine. Lab Chip.

[163] Rodriguez A.D., Horowitz L.F., Castro K., Kenerson H., Bhattacharjee N., Gandhe G., Raman A., Monnat R.J., Yeung R., Rostomily R.C. (2020). A microfluidic platform for functional testing of cancer drugs on intact tumor slices. Lab Chip.

[164] Chang T.C., Mikheev A.M., Huynh W., Monnat R.J., Rostomily R.C., Folch A. (2014). Parallel microfluidic chemosensitivity testing on individual slice cultures. Lab Chip.

[165] Mazzocchi A.R., Rajan S.A.P., Votanopoulos K.I., Hall A.R., Skardal A. (2018). In vitro patient-derived 3D mesothelioma tumor organoids facilitate patient-centric therapeutic screening. Sci. Rep..

[166] Tsai M., Kita A., Leach J., Rounsevell R., Huang J.N., Moake J., Ware R.E., Fletcher D.A., Lam W.A. (2012). In vitro modeling of the microvascular occlusion and thrombosis that occur in hematologic diseases using microfluidic technology. J. Clin. Investig..

[167] Bein A., Shin W., Jalili-Firoozinezhad S., Park M.H., Sontheimer-Phelps A., Tovaglieri A., Chalkiadaki A., Kim H.J., Ingber D.E. (2018). Microfluidic Organ-on-a-Chip Models of Human Intestine. Cell Mol. Gastroenterol. Hepatol..

[168] Gumuscu B., Albers H.J., van den Berg A., Eijkel J.C.T., van der Meer A.D. (2017). Compartmentalized 3D Tissue Culture Arrays under Controlled Microfluidic Delivery. Sci. Rep..

[169] Biglari S., Le T.Y.L., Tan R.P., Wise S.G., Zambon A., Codolo G., De Bernard M., Warkiani M., Schindeler A., Naficy S. (2019). Simulating Inflammation in a Wound Microenvironment Using a Dermal Wound-on-a-Chip Model. Adv. Health Mater..

[170] Ejiugwo M., Rochev Y., Gethin G., O’Connor G. (2021). Toward Developing Immunocompetent Diabetic Foot Ulcer-on-a-Chip Models for Drug Testing. Tissue Eng. Part C Methods.

[171] Ozdogan C.Y., Kenar H., Davun K.E., Yucel D., Doger E., Alagoz S. (2020). An in vitro 3D diabetic human skin model from diabetic primary cells. Biomed. Mater..

[172] Mascharak S., desJardins-Park H.E., Longaker M.T. (2020). Fibroblast Heterogeneity in Wound Healing: Hurdles to Clinical Translation. Trends Mol. Med..

[173] Maione A.G., Smith A., Kashpur O., Yanez V., Knight E., Mooney D.J., Veves A., Tomic-Canic M., Garlick J.A. (2016). Altered ECM deposition by diabetic foot ulcer-derived fibroblasts implicates fibronectin in chronic wound repair. Wound Repair Regen..

[174] Maione A.G., Brudno Y., Stojadinovic O., Park L.K., Smith A., Tellechea A., Leal E.C., Kearney C.J., Veves A., Tomic-Canic M. (2015). Three-dimensional human tissue models that incorporate diabetic foot ulcer-derived fibroblasts mimic in vivo features of chronic wounds. Tissue Eng. Part C Methods.

[175] Kim J.H., Martins-Green M. (2019). Protocol to Create Chronic Wounds in Diabetic Mice. J. Vis. Exp..

[176] Wang E.C.E., Higgins C.A. (2020). Immune cell regulation of the hair cycle. Exp. Dermatol..

[177] Nilforoushzadeh M., Rahimi Jameh E., Jaffary F., Abolhasani E., Keshtmand G., Zarkob H., Mohammadi P., Aghdami N. (2017). Hair Follicle Generation by Injections of Adult Human Follicular Epithelial and Dermal Papilla Cells into Nude Mice. Cell J..

[178] Asakawa K., Toyoshima K.E., Ishibashi N., Tobe H., Iwadate A., Kanayama T., Hasegawa T., Nakao K., Toki H., Noguchi S. (2012). Hair organ regeneration via the bioengineered hair follicular unit transplantation. Sci. Rep..

[179] Kageyama T., Yoshimura C., Myasnikova D., Kataoka K., Nittami T., Maruo S., Fukuda J. (2018). Spontaneous hair follicle germ (HFG) formation in vitro, enabling the large-scale production of HFGs for regenerative medicine. Biomaterials.

[180] Pang Q., Lou D., Li S., Wang G., Qiao B., Dong S., Ma L., Gao C., Wu Z. (2020). Smart Flexible Electronics-Integrated Wound Dressing for Real-Time Monitoring and On-Demand Treatment of Infected Wounds. Adv. Sci..

[181] Liu H., Wang Y., Cui K., Guo Y., Zhang X., Qin J. (2019). Advances in Hydrogels in Organoids and Organs-on-a-Chip. Adv. Mater..

[182] Hoyle N.P., Seinkmane E., Putker M., Feeney K.A., Krogager T.P., Chesham J.E., Bray L.K., Thomas J.M., Dunn K., Blaikley J. (2017). Circadian actin dynamics drive rhythmic fibroblast mobilization during wound healing. Sci. Transl. Med..

[183] Gao X., Wu L., O’Neil R.G. (2003). Temperature-modulated diversity of TRPV4 channel gating: Activation by physical stresses and phorbol ester derivatives through protein kinase C-dependent and -independent pathways. J. Biol. Chem..

